# Using a k-means clustering to identify novel phenotypes of acute ischemic stroke and development of its Clinlabomics models

**DOI:** 10.3389/fneur.2024.1366307

**Published:** 2024-03-27

**Authors:** Yao Jiang, Yingqiang Dang, Qian Wu, Boyao Yuan, Lina Gao, Chongge You

**Affiliations:** ^1^Laboratory Medicine Center, The Second Hospital and Clinical Medical School, Lanzhou University, Lanzhou, China; ^2^Department of Neurology, The Second Hospital and Clinical Medical School, Lanzhou University, Lanzhou, China

**Keywords:** acute ischemic stroke, novel phenotypes, machine learning, clustering algorithms, Clinlabomics models

## Abstract

**Objective:**

Acute ischemic stroke (AIS) is a heterogeneous condition. To stratify the heterogeneity, identify novel phenotypes, and develop Clinlabomics models of phenotypes that can conduct more personalized treatments for AIS.

**Methods:**

In a retrospective analysis, consecutive AIS and non-AIS inpatients were enrolled. An unsupervised k-means clustering algorithm was used to classify AIS patients into distinct novel phenotypes. Besides, the intergroup comparisons across the phenotypes were performed in clinical and laboratory data. Next, the least absolute shrinkage and selection operator (LASSO) algorithm was used to select essential variables. In addition, Clinlabomics predictive models of phenotypes were established by a support vector machines (SVM) classifier. We used the area under curve (AUC), accuracy, sensitivity, and specificity to evaluate the performance of the models.

**Results:**

Of the three derived phenotypes in 909 AIS patients [median age 64 (IQR: 17) years, 69% male], in phenotype 1 (*N* = 401), patients were relatively young and obese and had significantly elevated levels of lipids. Phenotype 2 (*N* = 463) was associated with abnormal ion levels. Phenotype 3 (*N* = 45) was characterized by the highest level of inflammation, accompanied by mild multiple-organ dysfunction. The external validation cohort prospectively collected 507 AIS patients [median age 60 (IQR: 18) years, 70% male]. Phenotype characteristics were similar in the validation cohort. After LASSO analysis, Clinlabomics models of phenotype 1 and 2 were constructed by the SVM algorithm, yielding high AUC (0.977, 95% CI: 0.961–0.993 and 0.984, 95% CI: 0.971–0.997), accuracy (0.936, 95% CI: 0.922–0.956 and 0.952, 95% CI: 0.938–0.972), sensitivity (0.984, 95% CI: 0.968–0.998 and 0.958, 95% CI: 0.939–0.984), and specificity (0.892, 95% CI: 0.874–0.926 and 0.945, 95% CI: 0.923–0.969).

**Conclusion:**

In this study, three novel phenotypes that reflected the abnormal variables of AIS patients were identified, and the Clinlabomics models of phenotypes were established, which are conducive to individualized treatments.

## Introduction

Acute ischemic stroke (AIS) is a highly heterogeneous disease characterized by a high risk of morbidity, disability, recurrence, and mortality (1, 2). It has been reported that the number of IS-related deaths is expected to increase further from 3.29 million in 2019 to 4.90 million by 2030 (3). Administration of antiplatelet and statin drugs in AIS patients is recommended by the American Heart Association (AHA) to reduce the risk of stroke recurrence and cardiovascular events (4). However, despite patients following the therapies of the guidelines, there is a substantial risk of recurrent stroke in AIS patients (5). A major barrier to intervention is the high heterogeneity of AIS. Therefore, stratifying the heterogeneity of AIS using multiple features can identify undescribed phenotypes that may respond differently to medication, making it possible to offer more personalized treatment to AIS patients. Recently, Ding et al. (6, 7) used unsupervised clustering algorithms to identify novel phenotypes with distinct traits in non-cardioembolic ischemic stroke (NCIS). Similarly, Chen et al. (8) and Schütz et al. (9) used the latent class analysis method to reveal the potential phenotypes of ischemic stroke with obstructive sleep apnea (OSA). Likewise, Lattanzi et al. (10) adopted the hierarchical cluster analysis to distinguish clinical phenotypes of the embolic stroke of an undetermined source. These studies elucidate the new tendency to discover potential phenotypes by understanding the heterogeneity of diseases based on a clustering algorithm.

The k-means clustering, as an unsupervised learning algorithm, can classify unlabeled data by maximizing the heterogeneity within different phenotypes (11) and also can identify similarities of potential phenotypes in a dataset (12). A large body of research work has shown that the k-means clustering algorithm can be used to reveal novel phenotypes of stroke (13), sepsis (14, 15), early-onset Alzheimer's disease (16), postoperative delirium symptoms (17), and coronary heart disease (CHD) (18), which can help to understand the potential pathogenesis and treatment respondence of diseases. For instance, with the availability of laboratory data, Guo et al. (15) used k-means clustering to categorize sepsis phenotype, reflecting the severity of sepsis and treatment effects. Similarly, Sriprasert et al. (18) classified postmenopausal women into different phenotypes based on nine metabolic laboratory indicators, revealing the relationship of subtypes to subclinical atherosclerosis.

Although clinical laboratories produce large amounts of laboratory results each day to assist clinical diagnosis (19), these data are not fully utilized (20). Hence, Wen et al. proposed a concept of clinical laboratory omics (Clinlabomics) using machine learning (ML) or deep learning algorithms to establish models based on clinical and laboratory data that can reveal valuable information hidden in a great deal of data (20).

Therefore, the objectives of this study were to investigate novel phenotypes of AIS patients based on clinical and laboratory data using a k-means clustering algorithm and maximizing the heterogeneity, compare the differences among phenotypes based on demographic, clinical, individual traits, physiological indices, and laboratory data, develop Clinlabomics models of AIS phenotypes, and evaluate the diagnostic performance of models, which have not been done previously.

## Methods

### Study design and population

This study consecutively enrolled AIS inpatients attending Lanzhou University Second Hospital between Dec 2019 and Dec 2022. Furthermore, we also prospectively collected AIS patients from January 2023 to January 2024 as an external validation dataset. The inclusion criteria were as follows: (1) age ≥18 years old; (2) first-ever AIS at admission within 24 h. Patients were excluded for malignant tumors, mental conditions, autoimmune diseases, intracranial hemorrhage, infection within 2 weeks before the onset of stroke, recurrent stroke, transient ischemic attacks (TIA), treated with anticoagulation or reperfusion, or missing data >5%. AIS, as defined by the World Health Organization (WHO), is a clinical syndrome with rapidly developing neurological deficit due to cerebrovascular cause, persisting for more than 24 h or death (21). The AIS was confirmed by computed tomography (CT) scan or diffusion weight imaging (DWI) on admission. Further, we also included a control group with 484 inpatients without any type of current or prior cerebral infarction but possessing clinical manifestations similar to AIS patients. This study was approved by the Ethics Committee of the Lanzhou University Second Hospital (IRB number: 2022A-710). Informed consent was obtained from all participants.

### Clinical and laboratory data collection

Medical records provided routinely available clinical data, including demographic data (age, gender, nationality, education, marriage), individual traits (height, weight, body mass index), vascular risk factors (the history of hypertension, diabetes, atrial fibrillation, coronary disease, and unhealthy habits including smoking and drinking), physiological indices (heart rate, oxygen saturation, blood pressure), the National Institutes of Health Stroke Scale (NIHSS) score that evaluates the stroke severity, Glasgow coma scale (GCS) that determines the degree of coma, modified Rankin scale (mRS) that assesses the degree of disability caused by stroke, Trial of Org 10172 in Acute Stroke Treatment (TOAST) classification that classifies etiological subtypes, and CT or DWI results that confirm the location and numbers of lesions. Based on the NIHSS score, scores of 1–4, 5–15, 16–20, and 21–42 were regarded as mild, moderate, moderate-to-severe, and severe stroke, respectively (22). An experienced senior neurologist (BY) examined and verified the NIHSS score, GCS, mRS, and TOAST classification in all included patients. There was a green channel for patients suspected of AIS, whose blood collection and detection were conducted immediately upon admission. In general, the results of complete blood count (CBC), biochemical tests, and coagulation examinations needed to be reported in 10, 30, and 30 min, respectively. Laboratory test results on admission were collected from the laboratory information system (LIS).

### Variable selection

In total, we collected data on 97 variables, where 76 variables could be measured, detected, or calculated. The calculation formula of inflammatory biomarkers was as follows: neutrophil to lymphocyte ratio (NLR) = neutrophil (NEU)/lymphocyte (LYM); lymphocyte to monocyte ratio (LMR) = LYM/ monocyte (MON); monocyte to high-density lipoprotein-cholesterol ratio (MHR) = MON/ high-density lipoprotein-cholesterol (HDL-C) (23); neutrophil to high-density lipoprotein-cholesterol ratio (NHR) = NEU/HDL-C (23); systemic immune-inflammation index (SII) = platelet (PLT) × NLR (24); system inflammation response index (SIRI) = NUE × MON/LYM (24); multi-inflammatory index 1 (MII-1) = NLR × C-reaction protein (CRP) (25); multi-inflammatory index 2 (MII-2)=PLT/LYM × CRP (25); multi-inflammatory index 3 (MII-3) = (PLT × NLR) × CRP (25); red blood cell distribution width to platelet ratio (RPR) = red blood cell distribution width coefficient of variation (RDWCV)/PLT (26). Additionally, we used the ln [total triglyceride (TG) (mg/dL) × fasting blood glucose (FBG) (mg/dL)/2] formula to calculate the triglyceride-glucose (TyG) index (27). The corresponding lipid parameters of the atherogenic index of plasma (AIP), lipoprotein combine index (LCI), non-high-density lipoprotein-cholesterol (non-HDL-C), atherogenic coefficient (AC), Castelli's index-I (CRI-I), and Castelli's index-II (CRI-II) were calculated by lg (TG/HDL-C) (28), total cholesterol (TC) × TG × low-density lipoprotein-cholesterol (LDL-C)/HDL-C (29), TC–HDL-C (30), non-HDL-C/HDL-C (31), TC/HDL-C (31), and LDL-C/HDL-C (31), respectively. We classified the 76 variables into 11 domains according to their commonality, including non-invasive physiological indices, individual characteristics, inflammatory biomarkers, red blood cell-related parameters, lipid parameters, diabetes-related biomarkers, renal function indicators, ions, liver function-related indicators, myocardial injury markers, and coagulative markers. Categorical variables, such as gender and stroke severity, were excluded because of the requirements of clustering analysis.

### Statistical analyses

A normal distribution of data was determined by the Kolmogorov-Smirnov test. The use of frequency counts and proportions (*n*%) expressed categorical variables that were compared using the Chi-square test and Fisher's exact test, if appropriate. Mean and standard deviation (SD), namely mean ± SD, was used to express normally distributed continuous variables, which were compared by a *t*-test. In contrast, non-normally distributed continuous variables were presented using median and interquartile range (IQR), namely M (Q1 - Q3), and compared by the Mann–Whitney *U*-test. The k-means clustering algorithm was used to identify novel phenotypes of AIS patients, where the optimal k was determined by the elbow method (32). The original data was transformed into standardized values (mean = 0, SD = 1) for clustering analysis. This clustering algorithm can partition observations into k clusters by assigning each observation to the nearest centroid (33). Once determined the phenotypes of AIS, we performed intergroup comparisons for the identification of significantly different variables. Further, a chord diagram was used to visualize abnormal variables classified by phenotype.

Before constructing models, we used the least absolute shrinkage and selection operator (LASSO) algorithm to perform variable selection for eliminating high multicollinearity variables (34). Subsequently, we used a random sampling method to divide patients in a 7:3 ratio into training and testing datasets. Next, a support vector machines (SVM) classifier was adopted to establish Clinlabomics predictive models, also regarded as phenotype classifiers, of AIS novel phenotypes. The SVM algorithm, which performs perfectly in dealing with both linear and non-linear data, can project training datasets into a multidimensional space, using a hyperplane to classify data (35), thus avoiding the overfitting problem (36). Receiver operating characteristic curves (ROC) were used to determine the optimal cut-off values of models, and the predictive performance of models was assessed by area under the receiver operating characteristic curve (AUC), accuracy, sensitivity, specificity, positive predictive value (PPV), and negative predictive value (NPV). All statistical analyses were performed on RStudio software (R version 4.3.0). A two-tailed *p* < 0.05 was regarded as statistical significance.

## Results

### Baseline characteristics of the study population

**Figure 1:**
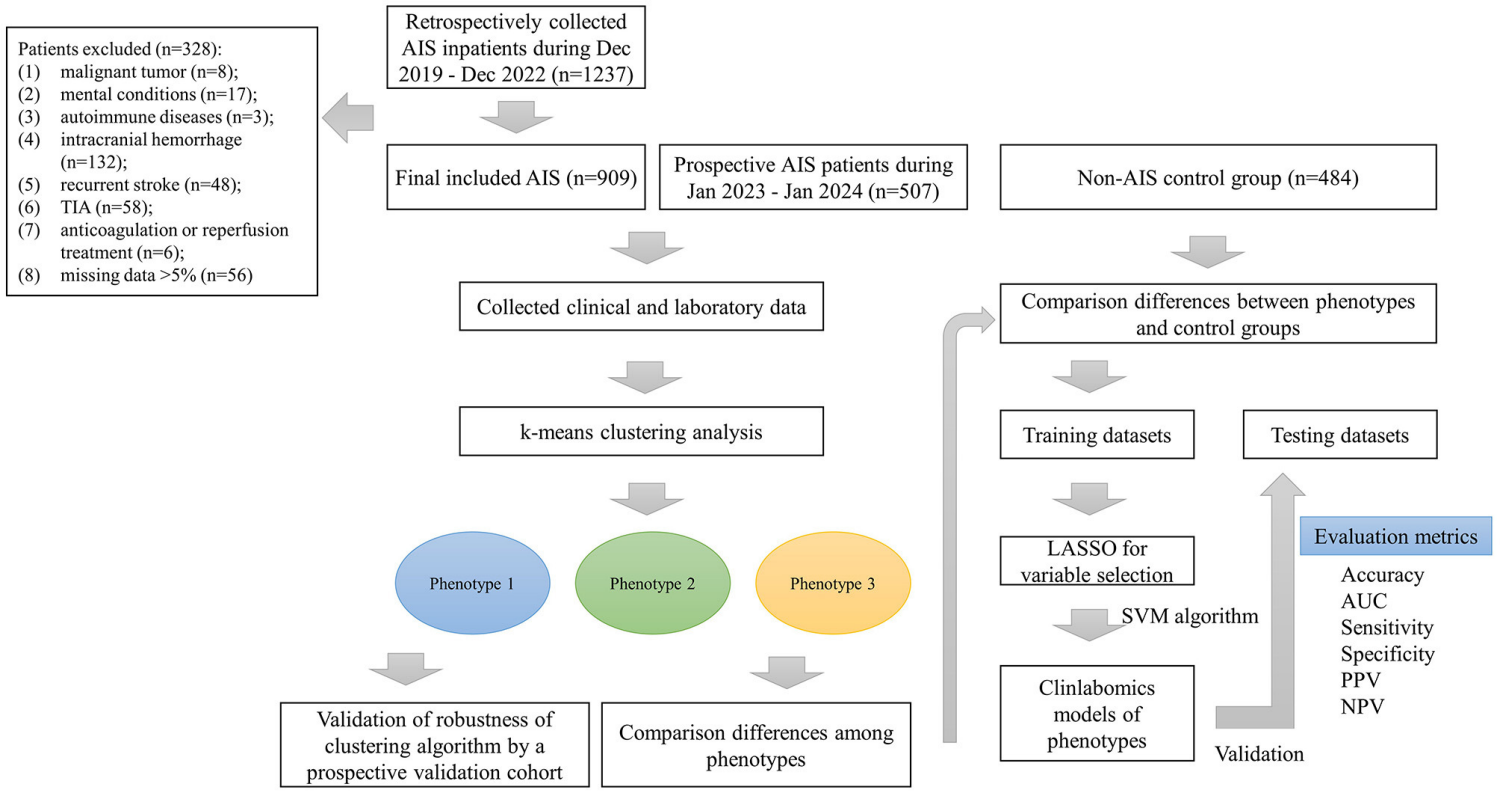
The patient selection process and flow chart. AIS, acute ischemic stroke; TIA, transient ischemic attack; LASSO, the least absolute shrinkage and selection operator; AUC, the area under curve; PPV, positive predictive value; NPV, negative predictive value.

In total, we retrospectively included 909 AIS patients [median age: 64 (IQR: 17) years, 69% male] and 484 non-AIS subjects [median age: 66 (IQR: 15) years, 53% male]. In addition, we also prospectively collected 507 AIS patients [median age 60 (IQR: 18) years, 70% male] as validation dataset to verify the robustness of the k-means clustering algorithm. Figure 1 shows the detailed patient selection process and flow chart of this study. Table 1 summarizes the characteristics of the participants. There were no significant differences in age, nationality, marriage, history of atrial fibrillation (AF) and CHD, heart rate (HR), oxygen saturation in arterial blood (SaO_2_), body mass index (BMI), mean corpuscular hemoglobin (MCH), RDWCV, TC, LDL-C, non-HDL-C, urea, urea to creatinine ratio (UCR), calcium (Ca), total bilirubin (TBIL), indirect bilirubin (IBIL), aspartate aminotransferase (AST), albumin (ALB), creatine kinase (CK), international normalized ratio (INR), thrombin time (TT), and fibrin degradation products (FDP) between the two groups (all *p* > 0.05).

**Table 1:** Baseline characteristics of included participants in the retrospective cohort.

**Variables**	**All participants (*n =* 1,393)**	**AIS group (*n =* 909)**	**Non-AIS group (*n =* 484)**	***p-*value**
**Demographic characteristics**				
Age	65 (55, 72)	64 (55, 72)	66 (57, 72)	0.172
**Gender**				
Female (%)	507 (36)	281 (31)	226 (47)	< 0.001
Male (%)	886 (64)	628 (69)	258 (53)	
**Nationality**				
Han (%)	1,294 (93)	845 (93)	449 (93)	0.982
Minority (%)	99 (7)	64 (7)	35 (7)	
**Marriage**				
Married (%)	1,374 (99)	893 (98)	481 (99)	0.132
Other status (%)	19 (1)	16 (2)	3 (1)	
**Education**				
High school diploma or higher (%)	473 (34)	285 (31)	188 (39)	0.006
Others (%)	920 (66)	624 (69)	296 (61)	
**Previous history**				
**HTN**				
No (%)	665 (48)	398 (44)	267 (55)	< 0.001
Yes (%)	728 (52)	511 (56)	217 (45)	
**AF**				
No (%)	1,373 (99)	892 (98)	481 (99)	0.103
Yes (%)	20 (1)	17 (2)	3 (1)	
**CHD**				
No (%)	1,330 (95)	873 (96)	457 (94)	0.212
Yes (%)	63 (5)	36 (4)	27 (6)	
**DM**				
No (%)	1,137 (82)	720 (79)	417 (86)	0.002
Yes (%)	256 (18)	189 (21)	67 (14)	
**Unhealthy habits**				
**Smoking**				
No (%)	1,140 (82)	706 (78)	434 (90)	< 0.001
Yes (%)	253 (18)	203 (22)	50 (10)	
No (%)	1,297 (93)	832 (92)	465 (96)	0.002
Yes (%)	96 (7)	77 (8)	19 (4)	
**Non-invasive physiological indices**				
HR (bpm)	77 (70, 86)	77 (70, 86)	77 (70, 85)	0.497
SBP (mmHg)	137 (123, 151)	140 (127, 156)	128 (119, 142)	< 0.001
DBP (mmHg)	80 (71, 89)	82 (73, 91)	76 (70, 84)	< 0.001
SaO_2_ (%)	96 (94, 96)	96 (94, 96)	95 (94, 96)	0.213
**Individual characteristics**				
Weight (Kg)	67 (60, 75)	68 (60, 75)	65 (59, 74)	0.001
Height (cm)	167 (160, 172)	168 (160, 172)	165 (160, 170)	< 0.001
BMI (Kg/m^2^)	24.22 (22.32, 26.22)	24.34 (22.41, 26.26)	24.16 (22.26, 26.08)	0.168
**Inflammatory biomarkers**				
WBC (10^9^/L)	6.16 (5.10, 7.66)	6.54 (5.40, 8.20)	5.60 (4.61, 6.66)	< 0.001
NEU (10^9^/L)	3.75 (2.86, 5.01)	4.25 (3.18, 5.56)	3.14 (2.46, 3.91)	< 0.001
LYM (10^9^/L)	1.63 (1.28, 2.06)	1.59 (1.25, 2.01)	1.70 (1.38, 2.10)	0.002
MON (10^9^/L)	0.44 (0.35, 0.56)	0.45 (0.36, 0.58)	0.42 (0.33, 0.52)	< 0.001
NLR	2.23 (1.64, 3.34)	2.55 (1.83, 3.77)	1.85 (1.38, 2.46)	< 0.001
LMR	3.72 (2.79, 4.82)	3.50 (2.61, 4.67)	4.05 (3.14, 5.14)	< 0.001
MHR	0.43 (0.31, 0.58)	0.46 (0.33, 0.61)	0.38 (0.29, 0.53)	< 0.001
NHR	3.65 (2.63, 5.24)	4.16 (3.04, 5.98)	2.89 (2.14, 3.90)	< 0.001
SII (10^9^/L)	413 (281, 667)	487 (323, 748)	330 (232, 472)	< 0.001
SIRI (10^9^/L)	1.01 (0.65, 1.64)	1.16 (0.78, 1.92)	0.79 (0.51, 1.13)	< 0.001
MII-1	6.27 (2.43, 13.05)	7.98 (2.61, 16.33)	5.00 (2.07, 7.89)	< 0.001
MII-2	205 (88, 602)	341 (115, 721)	115 (68, 283)	< 0.001
MII-3	1,115 (417, 2503)	1,413 (464, 3211)	841 (353, 1496)	< 0.001
RPR	0.09 (0.06, 1.12)	0.07 (0.06, 0.09)	3.70 (1.01, 3.70)	< 0.001
CRP (mg/L)	0.90 (0.09, 5.47)	2.84 (0.99, 5.96)	0.07 (0.06, 0.09)	< 0.001
**Red blood cell-related parameters**				
RBC (10^12^/L)	4.72 (4.35, 5.11)	4.78 (4.41, 5.16)	4.59 (4.29, 4.97)	< 0.001
HGB (g/L)	148 (136, 158)	149 (138, 160)	143 (133, 156)	< 0.001
HCT	0.44 (0.41, 0.47)	0.44 (0.41, 0.48)	0.43 (0.4, 0.46)	< 0.001
MCV (fL)	93.3 (90.0, 96.1)	92.9 (89.7, 95.9)	93.8 (90.9, 96.5)	< 0.001
MCH (pg)	31.3 (30.1, 32.4)	31.2 (30.1, 32.4)	31.3 (30.1, 32.4)	0.995
MCHC (g/L)	335 (327, 342)	336 (329, 342)	332 (325, 340)	< 0.001
RDWCV (%)	12.8 (12.3, 13.3)	12.8 (12.3, 13.4)	12.9 (12.4, 13.3)	0.623
**Lipid parameters**				
TC (mmol/L)	4.01 (3.33, 4.72)	3.99 (3.31, 4.70)	4.06 (3.36, 4.76)	0.386
TG (mmol/L)	1.38 (1.01, 1.87)	1.41 (1.04, 1.95)	1.31 (0.96, 1.77)	0.005
HDL-C (mmol/L)	1.03 (0.88, 1.22)	1.00 (0.85, 1.18)	1.10 (0.94, 1.27)	< 0.001
LDL-C (mmol/L)	2.66 (2.12, 3.21)	2.67 (2.13, 3.2)	2.66 (2.12, 3.24)	0.924
AIP	0.12 (-0.03, 0.28)	0.14 (0, 0.3)	0.08 (-0.08, 0.24)	< 0.001
LCI	13.96 (7.79, 25.81)	14.57 (7.99, 27.35)	12.91 (7.33, 21.99)	0.002
non-HDL-C (mmol/L)	2.96 (2.35, 3.61)	2.99 (2.37, 3.62)	2.91 (2.32, 3.58)	0.416
AC	2.87 (2.16, 3.58)	2.97 (2.27, 3.71)	2.67 (2.00, 3.37)	< 0.001
CRI-I	3.87 (3.16, 4.58)	3.97 (3.27, 4.71)	3.67 (3.00, 4.37)	< 0.001
CRI-II	2.59 (1.99, 3.15)	2.69 (2.08, 3.27)	2.42 (1.89, 3.02)	< 0.001
**Diabetes-related biomarkers**				
GLU (mmol/L)	5.44 (4.77, 7.09)	5.89 (4.93, 7.98)	5.02 (4.58, 5.90)	< 0.001
TyG	8.74 (8.36, 9.22)	8.85 (8.41, 9.35)	8.61 (8.24, 8.97)	< 0.001
**Renal function indicators**				
Urea (mmol/L)	5.6 (4.5, 6.8)	5.7 (4.5, 6.9)	5.5 (4.6, 6.7)	0.192
CREA (μmol/L)	63.1 (52.7, 74.2)	63.9 (53.5, 75.3)	61.1 (50.9, 71. 7)	0.001
UCR	0.09 (0.07, 0.11)	0.08 (0.07, 0.10)	0.09 (0.07, 0.11)	0.053
UA (μmol/L)	308 (252, 371)	312 (254, 379)	299 (249, 355)	0.008
**Ion**				
K (mmol/L)	3.82 (3.57, 4.04)	3.79 (3.53, 4.01)	3.87 (3.64, 4.09)	< 0.001
NA (mmol/L)	140.1 (138.3, 142.0)	140.0 (138.0, 141.7)	141.0 (139.0, 142.2)	< 0.001
Cl (mmol/L)	106.0 (104.0, 108.0)	105.6 (103.0, 107.3)	106.7 (105.0, 108.1)	< 0.001
CO_2_ (mmol/L)	24.5 (22.8, 26.2)	24.3 (22.6, 26.0)	25.0 (23.4, 26.5)	< 0.001
Ca (mmol/L)	2.25 (2.18, 2.32)	2.25 (2.18, 2.32)	2.25 (2.18, 2.32)	0.585
P (mmol/L)	1.07 (0.94, 1.20)	1.04 (0.92, 1.18)	1.11 (0.98, 1.23)	< 0.001
Mg (mmol/L)	0.86 (0.81, 0.91)	0.85 (0.80, 0.90)	0.87 (0.83, 0.91)	< 0.001
**Liver function-related indicators**				
TBIL (μmol/L)	14.4 (11.0, 18.9)	14.8 (11.0, 19.5)	14.0 (11.1, 17.9)	0.118
DBIL (μmol/L)	2.8 (2.0, 3.8)	2.8 (2.0, 4.0)	2.7 (2.0, 3.6)	0.046
IBIL (μmol/L)	11.5 (8.7, 15.3)	11.7 (8.7, 15.6)	11.1 (8.7, 14.7)	0.254
ALT (U/L)	18 (13, 27)	18 (12, 26)	19 (13, 28)	0.042
AST (U/L)	22 (18, 27)	22 (18, 27)	22 (18, 27)	0.85
AAR	1.17 (0.89, 1.55)	1.18 (0.92, 1.60)	1.14 (0.86, 1.47)	0.006
GGT (U/L)	24 (16, 36)	24 (17, 38)	22 (16, 34)	0.002
ALP (U/L)	84 (70, 102)	87 (72, 105)	80 (67, 94)	< 0.001
CHE (U/mL)	7.8 ± 1.55	7.87 ± 1.61	7.68 ± 1.43	0.028
TP (g/L)	66.9 (62.6, 71.4)	67.3 (62.8, 71.6)	66.1 (62.1, 70.9)	0.005
ALB (g/L)	39.8 (37.4, 42.4)	39.8 (37.4, 42.3)	39.9 (37.5, 42.8)	0.295
GLB (g/L)	26.9 (23.8, 30.1)	27.3 (24.1, 30.7)	26.0 (23.5, 29.1)	< 0.001
AGR	1.49 (1.32, 1.68)	1.46 (1.29, 1.66)	1.54 (1.40, 1.71)	< 0.001
**Myocardial injury marker**				
CK (U/L)	73 (51, 101)	73 (51, 103)	72 (53, 100)	0.644
CK-MB (U/L)	12 (10, 15)	12 (10, 15)	12 (10, 14)	0.016
LDH (U/L)	189 (165, 220)	193 (166, 223)	184 (161, 211)	< 0.001
**Coagulative markers**				
PT (s)	11.1 (10.6, 11.7)	11.2 (10.7, 11.8)	11 (10.5, 11.4)	< 0.001
PTA (%)	99 (91, 106)	97 (90, 105)	100 (93, 107)	< 0.001
INR	1.00 (0.96, 1.05)	1.00 (0.96, 1.05)	1.00 (0.96, 1.04)	0.563
APTT (s)	30.8 (28.7, 33.1)	30.5 (28.5, 33.1)	31.2 (29.0, 33.2)	0.039
FIB (g/L)	2.97 (2.61, 3.40)	3.03 (2.67, 3.48)	2.89 (2.51, 3.24)	< 0.001
TT (s)	14.1 (13.3, 14.9)	14.1 (13.3, 14.9)	14.3 (13.5, 15.0)	0.063
DD (μg/mL)	0.39 (0.22, 0.72)	0.41 (0.23, 0.78)	0.37 (0.22, 0.63)	0.03
FDP (μg/mL)	1.03 (0.62, 1.83)	1.04 (0.63, 2.00)	1.01 (0.60, 1.72)	0.536

AIS, acute ischemic stroke; HTN, hypertension; AF, atrial fibrillation; CHD, coronary heart disease; DM, diabetes mellitus; HR, heart rate; SaO_2_, oxygen saturation in arterial blood; SBP, systolic blood pressure; DBP, diastolic blood pressures; BMI, body mass index; WBC, white blood cell; NEU, neutrophil; LYM, lymphocyte; MON, monocyte; NLR, neutrophil to lymphocyte ratio; LMR, lymphocyte to monocyte ratio; MHR, monocyte to high-density lipoprotein-cholesterol ratio; NHR, neutrophil to high-density lipoprotein-cholesterol ratio; SII, systemic immune-inflammation index; SIRI, system inflammation response index; MII-1, multi-inflammatory index-1; MII-2, multi-inflammatory index-2; MII-3, multi-inflammatory index-3; RPR, red blood cell distribution width to platelet ratio; CRP, C-reaction protein; RBC, red blood cell; HGB, hemoglobin; HCT, hematocrit; MCV, mean corpuscular volume; MCH, mean corpuscular hemoglobin; MCHC, mean corpuscular hemoglobin concentration; RDWSD, red blood cell distribution width standard deviation; RDWCV, red blood cell distribution width coefficient of variation; TC, total cholesterol; TG, total triglyceride; HDL-C, high-density lipoprotein-cholesterol; LDL-C, low-density lipoprotein cholesterol; AIP, atherogenic index of plasma; LCI, lipoprotein combine index; AC, atherogenic coefficient; CRI-I, Castelli's index-I; CRI-II, Castelli's index-II; non-HDL, non-high density lipoprotein-cholesterol; GLU, glucose; TyG, triglyceride-glucose; CREA, creatinine; UCR, urea to creatinine ratio; UA, uric acid; K, potassium; Na, sodium; Cl, chlorine; CO_2_, carbon dioxide; Ca, calcium; P, phosphorus; Mg, magnesium; TBIL, total bilirubin; DBIL, direct bilirubin; IBIL, indirect bilirubin; ALT, alanine transaminase; AST, aspartate aminotransferase; AAR, aspartate aminotransferase to alanine transaminase ratio; GGT, γ glutamyl transpeptadase; ALP, alkaline phosphatase; CHE, cholinesterase; TP, total protein; ALB, albumin; GLB, globulin; AGR, albumin to globulin ratio; CK, creatine kinase; CK-MB, creatine kinase-MB; LDH, lactic dehydrogenase; PT, prothrombin time; PTA, prothrombin activity; INR, international normalized ratio; APTT, activated partial thromboplastin time; FIB, fibrinogen; TT, thrombin time; FDP, fibrin degradation products; DD, D-Dimer.

### K-means clustering

**Figure 2:**
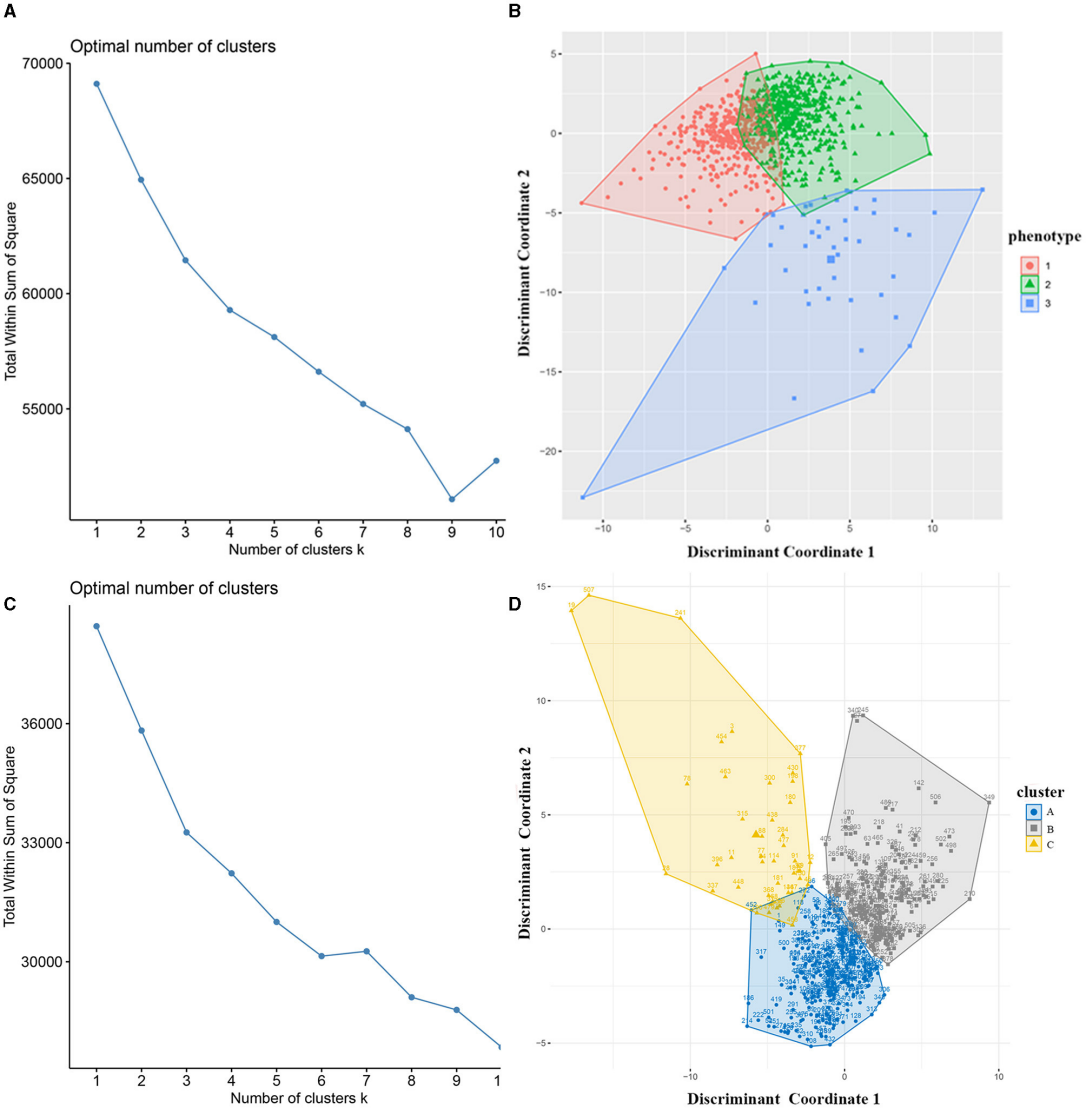
Identification of phenotypes of AIS patients using k-means clustering. **(A)** The optimal k value was determined using the elbow method; **(B)** Plotting of individual observations of each phenotype in discriminant component space; **(C)** The optimal k value in the validation cohort; **(D)** Individual observations of each cluster in discriminant component space in the validation dataset. AIS, acute ischemic stroke.

**Figure 3:**
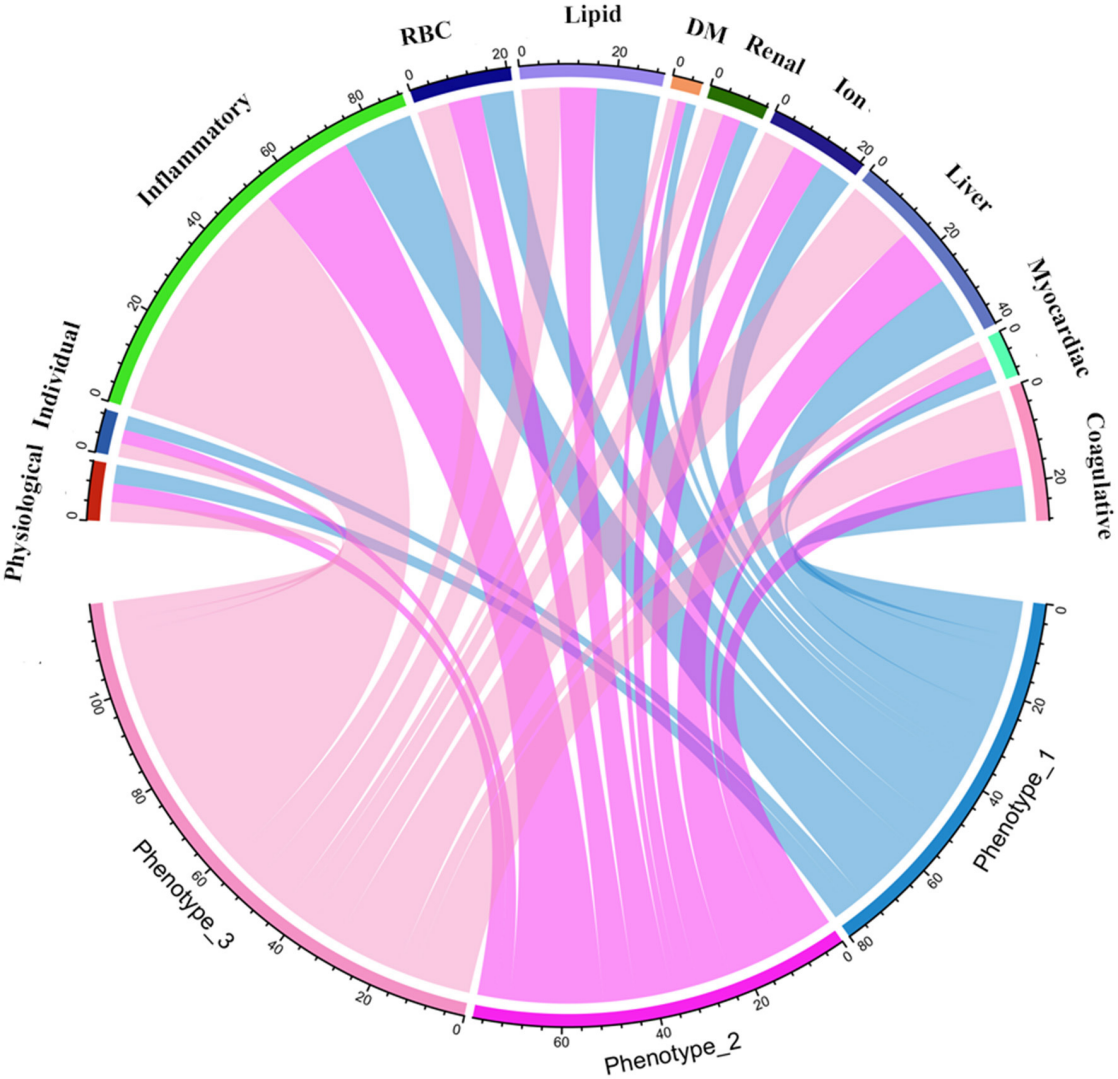
Chord diagrams show the relationships between phenotypes and 11 domains. RBC, red blood cell; DM, diabetes mellitus.

We used the elbow method to determine the optimal k value of 3 (Figure 2A) and divided 909 AIS patients into three novel phenotypes (Figure 2B). Figure 3 describes the abnormal variables of three phenotypes. Patients in phenotype 1 (*n* = 401) were relatively young and obese and had significantly elevated levels of lipids. Phenotype 2 (*n* = 463) was associated with abnormal ion levels. Phenotype 3 (*n* = 45) was characterized by the highest level of inflammation, accompanied by mild multiple-organ dysfunction. Table 2 compares the statistical difference among phenotypes in demographic, clinical characteristics, and laboratory data.

**Table 2:** Characteristics of three phenotypes based on the k-means clustering analysis.

**Variables**	**Total (*n =* 909)**	**Phenotypes**			***p***
		**Phenotype-1 (*****n** =* **401)**	**Phenotype-2 (*****n** =* **463)**	**Phenotype-3 (*****n** =* **45)**	
**Demographic characteristics**					
Age	64 (55, 72)	61 (53, 70)	66 (56, 74)	70 (54, 75)	< 0.001^a^; 0.017^b^; 0.591
**Gender**					
Female (%)	281 (31)	120 (30)	147 (32)	14 (31)	0.614; 1; 1
Male (%)	628 (69)	281 (70)	316 (68)	31 (69)	
**Nationality**					
Han (%)	845 (93)	377 (94)	426 (92)	42 (93)	0.558; 0.746; 1
Minority (%)	64 (7)	24 (6)	37 (8)	3 (7)	
**Marriage**					
Married (%)	893 (98)	391 (98)	457 (99)	45 (100)	0.31; 0.608; 1
Other status (%)	16 (2)	10 (2)	6 (1)	0 (0)	
**Education**					
High school diploma or higher (%)	285 (31)	122 (30)	151 (33)	12 (27)	0.537; 0.726; 0.517
Others (%)	624 (69)	279 (70)	312 (67)	33 (73)	
**Clinical classification and scores**					
**TOAST**					
LAA (%)	365 (40)	149 (37)	196 (42)	20 (44)	0.105; 0.1; 0.361
SAO (%)	267 (29)	134 (33)	125 (27)	8 (18)	
Others (%)	277 (31)	118 (30)	142 (31)	17 (38)	
**Scales**					
NIHSS	3 (1, 5)	3 (1, 6)	2 (1, 5)	11 (6, 16)	0.058; < 0.001^b^; < 0.001^c^
GCS	15 (15, 15)	15 (15, 15)	15 (15, 15)	13 (9, 15)	0.004^a^; < 0.001^b^; < 0.001^c^
**mRS**					
0–2 (%)	515 (57)	234 (58)	275 (59)	6 (13)	0.81; < 0.001^b^; < 0.001^c^
3–6 (%)	394 (43)	167 (42)	188 (41)	39 (87)	
**Previous history**					
**HTN**					
No (%)	398 (44)	162 (40)	213 (46)	23 (51)	0.112; 0.221; 0.618
Yes (%)	511 (56)	239 (60)	250 (54)	22 (49)	
**AF**					
No (%)	892 (98)	396 (99)	453 (98)	43 (96)	0.445; 0.151; 0.288
Yes (%)	17 (2)	5 (1)	10 (2)	2 (4)	
**CHD**					
No (%)	873 (96)	390 (97)	440 (95)	43 (96)	0.133; 0.63; 1
Yes (%)	36 (4)	11 (3)	23 (5)	2 (4)	
**DM**					
No (%)	720 (79)	298 (74)	384 (83)	38 (84)	0.003^a^; 0.189; 0.961
Yes (%)	189 (21)	103 (26)	79 (17)	7 (16)	
**Unhealthy habits**					
**Smoking**					
No (%)	706 (78)	308 (77)	368 (79)	30 (67)	0.386; 0.186; 0.071
Yes (%)	203 (22)	93 (23)	95 (21)	15 (33)	
**Drinking**					
No (%)	832 (92)	365 (91)	430 (93)	37 (82)	0.382; 0.068; 0.02^c^
Yes (%)	77 (8)	36 (9)	33 (7)	8 (18)	
**MRI location**					
**Lesions**					
One site (%)	417 (46)	202 (50)	211 (46)	4 (9)	0.18; < 0.001^b^; < 0.001^c^
Multiple sites (%)	492 (54)	199 (50)	252 (54)	41 (91)	
**Carotid artery ultrasound**					
**IMT: Right**					
≤ 1.0 mm (%)	705 (78)	319 (80)	349 (75)	37 (82)	0.168; 0.82; 0.399
>1.0 mm (%)	204 (22)	82 (20)	114 (25)	8 (18)	
**IMT: Left**					
≤ 1.0 mm (%)	643 (71)	281 (70)	325 (70)	37 (82)	1; 0.125; 0.126
>1.0 mm (%)	266 (29)	120 (30)	138 (30)	8 (18)	
**CP**					
No (%)	248 (27)	100 (25)	138 (30)	10 (22)	0.128; 0.827; 0.37
Yes (%)	661 (73)	301 (75)	325 (70)	35 (78)	
**VP**					
None (%)	248 (27)	100 (25)	138 (30)	10 (22)	0.259; 0.386; 0.257
SP (%)	65 (7)	32 (8)	32 (7)	1 (2)	
VP (%)	596 (66)	269 (67)	293 (63)	34 (76)	
**CS**					
No (%)	798 (88)	351 (88)	411 (89)	36 (80)	0.648; 0.237; 0.137
Yes (%)	111 (12)	50 (12)	52 (11)	9 (20)	
**Non-invasive physiological indices**					
HR (bpm)	77 (70, 86)	78 (72, 88)	75 (68, 83)	86 (76, 100)	< 0.001^a^; < 0.001^b^; < 0.001^c^
SBP (mmHg)	140 (127, 156)	143 (130, 157)	138 (124, 152)	148 (140, 161)	< 0.001^a^; 0.148; 0.004^c^
DBP (mmHg)	82 (73, 91)	85 (76, 93)	79 (71, 88)	80 (73, 93)	< 0.001^a^; 0.296; 0.28
SaO_2_ (%)	96 (94, 96)	96 (94, 96)	96 (94, 96)	96 (94, 98)	0.682; 0.652; 0.785
**Individual characteristics**					
Weight (Kg)	68 (60, 75)	70 (64, 75)	65 (60, 74)	65 (60, 70)	< 0.001^a^; 0.003^b^; 0.38
Height (cm)	168 (160, 172)	168 (160, 172)	168 (160, 172)	170 (160, 174)	0.61; 0.513; 0.384
BMI (Kg/m^2^)	24.34 (22.41, 26.26)	25.06 (23.15, 27.34)	23.88 (22.04, 25.76)	22.86 (20.76, 24.8)	< 0.001^a^; < 0.001^b^; 0.073
**Inflammatory biomarkers**					
WBC (10^9^/L)	6.54 (5.40, 8.20)	6.94 (5.89, 8.46)	5.92 (4.98, 7.38)	12.00 (9.00, 15.32)	< 0.001^a^; < 0.001^b^; < 0.001^c^
NEU (10^9^/L)	4.25 (3.18, 5.56)	4.52 (3.54, 5.73)	3.76 (2.88, 4.95)	10.45 (7.27, 12.99)	< 0.001^a^; < 0.001^b^; < 0.001^c^
LYM (10^9^/L)	1.59 (1.25, 2.01)	1.75 (1.42, 2.21)	1.55 (1.17, 1.92)	0.86 (0.61, 1.29)	< 0.001^a^; < 0.001^b^; < 0.001^c^
MON (10^9^/L)	0.45 (0.36, 0.58)	0.45 (0.36, 0.59)	0.45 (0.36, 0.56)	0.64 (0.51, 1.04)	0.4; < 0.001^b^; < 0.001^c^
NLR	2.55 (1.83, 3.77)	2.47 (1.84, 3.57)	2.45 (1.75, 3.5)	9.8 (6.82, 19.98)	0.637; < 0.001^b^; < 0.001^c^
LMR	3.50 (2.61, 4.67)	3.90 (2.89, 5.07)	3.39 (2.59, 4.42)	1.40 (0.87, 1.74)	< 0.001^a^; < 0.001^b^; < 0.001^c^
MHR	0.46 (0.33, 0.61)	0.44 (0.32, 0.61)	0.46 (0.34, 0.59)	0.79 (0.56, 1.08)	0.5; < 0.001^b^; < 0.001^c^
NHR	4.16 (3.04, 5.98)	4.30 (3.34, 6.10)	3.81 (2.78, 5.30)	10.47 (7.76, 12.75)	< 0.001^a^; < 0.001^b^; < 0.001^c^
SII (10^9^/L)	487 (323, 748)	501 (341, 777)	439 (296, 678)	1945 (1222, 3016)	< 0.001^a^; < 0.001^b^; < 0.001^c^
SIRI (10^9^/L)	1.16 (0.78, 1.92)	1.12 (0.78, 1.8)	1.13 (0.74, 1.77)	8.57 (3.75, 16.43)	0.556; < 0.001^b^; < 0.001^c^
MII-1	7.98 (2.61, 16.33)	7.21 (2.73, 14.46)	7.67 (2.14, 14.99)	82.96 (24.98, 303.47)	0.702; < 0.001^b^; < 0.001^c^
MII-2	341 (115, 721)	325 (120, 679)	321 (98, 695)	1297 (301, 10385)	0.804; < 0.001^b^; < 0.001^c^
MII-3	1413 (464, 3211)	1509 (534, 2959)	1184 (339, 2906)	13549 (4224, 84269)	0.059; < 0.001^b^; < 0.001^c^
RPR	0.07 (0.06, 0.09)	0.06 (0.05, 0.07)	0.07 (0.06, 0.09)	0.08 (0.06, 0.11)	< 0.001^a^; < 0.001^b^; 0.738
CRP (mg/L)	2.84 (0.99, 5.96)	2.67 (1.09, 5.96)	2.79 (0.88, 5.96)	7.85 (2.14, 59.19)	0.568; < 0.001^b^; < 0.001^c^
**Red blood cell-related parameters**					
RBC (10^12^/L)	4.78 (4.41, 5.16)	4.96 (4.65, 5.32)	4.64 (4.25, 4.96)	4.33 (3.91, 4.99)	< 0.001^a^; < 0.001^b^; 0.017^c^
HGB (g/L)	149 (138, 160)	155 (145, 164)	145 (135, 155)	136 (117, 153)	< 0.001^a^; < 0.001^b^; 0.006^c^
HCT	0.44 (0.41, 0.48)	0.46 (0.43, 0.49)	0.43 (0.4, 0.46)	0.40 (0.35, 0.45)	< 0.001^a^; < 0.001^b^; 0.003^c^
MCV (fL)	92.9 (89.7, 95.9)	91.9 (89.1, 94.8)	93.6 (90.6, 96.9)	92.8 (89, 96.4)	< 0.001^a^; 0.476; 0.186
MCH (pg)	31.2 (30.1, 32.4)	31.0 (30.0, 32.1)	31.5 (30.3, 32.6)	31.4 (30.1, 32.8)	< 0.001^a^; 0.308; 0.67
MCHC (g/L)	336 (329, 342)	337 (330, 343)	335 (328, 342)	337 (325, 348)	0.012^a^; 0.81; 0.493
RDWCV (%)	12.8 (12.3, 13.4)	12.6 (12.0, 13.2)	13.0 (12.4, 13.5)	13.5 (12.7, 14.0)	< 0.001^a^; < 0.001^b^; 0.003^c^
**Lipid parameters**					
TC (mmol/L)	3.99 (3.31, 4.7)	4.7 (4.12, 5.33)	3.43 (2.99, 4)	3.59 (2.95, 4.13)	< 0.001^a^; < 0.001^b^; 0.512
TG (mmol/L)	1.41 (1.04, 1.95)	1.96 (1.55, 2.62)	1.13 (0.88, 1.4)	1.22 (0.8, 1.52)	< 0.001^a^; < 0.001^b^; 0.617
HDL-C (mmol/L)	1 (0.85, 1.18)	1.03 (0.89, 1.21)	0.98 (0.85, 1.15)	0.98 (0.72, 1.21)	0.011^a^; 0.135; 0.456
LDL-C (mmol/L)	2.67 (2.13, 3.2)	3.19 (2.76, 3.69)	2.29 (1.89, 2.67)	2.26 (1.73, 2.82)	< 0.001^a^; < 0.001^b^; 0.643
AIP	0.14 (0, 0.3)	0.28 (0.14, 0.44)	0.06 (-0.08, 0.17)	0.06 (-0.06, 0.25)	< 0.001^a^; < 0.001^b^; 0.347
LCI	14.57 (7.99, 27.35)	28.65 (19.13, 44.02)	8.93 (5.76, 13.34)	9.55 (4.96, 19.98)	< 0.001^a^; < 0.001^b^; 0.412
non-HDL-C (mmol/L)	2.99 (2.37, 3.62)	3.64 (3.12, 4.19)	2.46 (2.04, 2.95)	2.56 (1.95, 3.14)	< 0.001^a^; < 0.001^b^; 0.181
AC	2.97 (2.27, 3.71)	3.56 (2.96, 4.19)	2.45 (1.96, 3.09)	2.55 (1.97, 3.64)	< 0.001^a^; < 0.001^b^; 0.227
CRI-I	3.97 (3.27, 4.71)	4.56 (3.96, 5.19)	3.45 (2.96, 4.09)	3.55 (2.97, 4.64)	< 0.001^a^; < 0.001^b^; 0.227
CRI-II	2.69 (2.08, 3.27)	3.11 (2.65, 3.61)	2.29 (1.82, 2.83)	2.28 (1.84, 3.33)	< 0.001^a^; < 0.001^b^; 0.327
**Diabetes-related biomarkers**					
GLU (mmol/L)	5.89 (4.93, 7.98)	6.65 (5.35, 9.44)	5.34 (4.66, 6.64)	6.66 (5.09, 10.18)	< 0.001^a^; 0.8; < 0.001^c^
TyG	8.85 (8.41, 9.35)	9.32 (8.94, 9.75)	8.50 (8.21, 8.84)	8.70 (8.27, 9.22)	< 0.001^a^; < 0.001^b^; 0.015^c^
**Renal function indicators**					
Urea (mmol/L)	5.7 (4.5, 6.9)	5.6 (4.6, 6.9)	5.7 (4.5, 6.72)	6.8 (4.7, 9.0)	0.271; 0.007^b^; 0.002^c^
CREA (μmol/L)	63.9 (53.5, 75.3)	65.2 (55.6, 76.2)	62.5 (51.8, 73.6)	66.1 (50.9, 82.7)	0.008^a^; 0.892; 0.448
UCR	0.08 (0.07, 0.1)	0.08 (0.07, 0.11)	0.08 (0.07, 0.1)	0.09 (0.08, 0.12)	0.803; 0.028^b^; 0.034^c^
UA (μmol/L)	312.0 (254.0, 379.0)	328.0 (262.0, 406.0)	300.0 (246.5, 357.5)	303.0 (248.6, 393.0)	< 0.001^a^; 0.31; 0.431
**Ion**					
K (mmol/L)	3.79 (3.53, 4.01)	3.79 (3.51, 4.03)	3.80 (3.56, 3.99)	3.72 (3.53, 4.01)	0.724; 0.646; 0.421
NA (mmol/L)	140.0 (138.0, 141.7)	139.7 (138.0, 141.2)	140.4 (138.3, 142.0)	138.0 (135.3, 140.0)	< 0.001^a^; < 0.001^b^; < 0.001^c^
Cl (mmol/L)	105.6 (103.0, 107.3)	105.0 (102.0, 106.8)	106.0 (104.0, 108.0)	105.0 (102.0, 107.2)	< 0.001^a^; 0.634; 0.039^c^
CO_2_ (mmol/L)	24.3 (22.6, 26)	24.3 (22.5, 26.1)	24.4 (22.8, 26.1)	22.3 (19.5, 24.3)	0.574; < 0.001^b^; < 0.001^c^
Ca (mmol/L)	2.25 (2.18, 2.32)	2.29 (2.23, 2.37)	2.20 (2.14, 2.28)	2.23 (2.13, 2.29)	< 0.001^a^; < 0.001^b^; 0.932
P (mmol/L)	1.04 (0.92, 1.18)	1.03 (0.92, 1.17)	1.05 (0.93, 1.18)	1.03 (0.85, 1.15)	0.41; 0.522; 0.704
Mg (mmol/L)	0.85 (0.8, 0.9)	0.86 (0.81, 0.91)	0.84 (0.8, 0.89)	0.83 (0.76, 0.88)	< 0.001^a^; 0.015^b^; 0.219
**Liver function-related indicators**					
TBIL (μmol/L)	14.8 (11.0, 19.5)	14.5 (10.9, 18.3)	14.9 (11.0, 20.1)	17.4 (13.4, 23.4)	0.141; 0.008^b^; 0.049^c^
DBIL (μmol/L)	2.8 (2.0, 4.0)	2.5 (1.8, 3.4)	3.0 (2.2, 4.3)	3.5 (2.1, 6.3)	< 0.001^a^; < 0.001^b^; 0.057
IBIL (μmol/L)	11.7 (8.7, 15.6)	11.6 (9.0, 15.2)	11.6 (8.5, 15.9)	13.0 (9.6, 18.2)	0.968; 0.075; 0.088
ALT (U/L)	18 (12, 26)	19 (13, 27)	17 (11, 25)	17 (13, 26)	0.003^a^; 0.696; 0.336
AST (U/L)	22 (18, 27)	21 (19, 27)	21 (17, 25)	26 (22, 40)	0.11; < 0.001^b^; < 0.001^c^
AAR	1.18 (0.92, 1.60)	1.11 (0.88, 1.54)	1.22 (0.96, 1.58)	1.57 (1.24, 2.17)	0.005^a^; < 0.001^b^; < 0.001^c^
GGT (U/L)	24 (17, 38)	28 (20, 43)	21 (15, 32)	33 (19, 86)	< 0.001^a^; 0.339; < 0.001^c^
ALP (U/L)	87 (72, 105)	92 (76, 109)	83 (69, 98)	97 (67, 120)	< 0.001^a^; 0.899; 0.084
CHE (U/mL)	7.87 ± 1.61	8.81 ± 1.36	7.18 ± 1.31	6.60 ± 1.98	< 0.001^a^; < 0.001^b^; 0.062
TP (g/L)	67.4 ± 6.5	70.4 ± 6.3	64.8 ± 5.6	68.0 ± 6.7	< 0.001^a^; 0.027^b^; 0.003^c^
ALB (g/L)	39.8 (37.4, 42.3)	41.5 (39.4, 43.8)	38.8 (36.5, 40.9)	37.7 (34.1, 39.9)	< 0.001^a^; < 0.001^b^; 0.066
GLB (g/L)	27.3 (24.1, 30.7)	28.5 (25.9, 32.1)	25.7 (23.0, 28.8)	30.2 (27.6, 34.4)	< 0.001^a^; 0.013^b^; < 0.001^c^
AGR	1.46 (1.29, 1.66)	1.44 (1.28, 1.63)	1.49 (1.32, 1.71)	1.29 (1.03, 1.39)	0.004^a^; < 0.001^b^; < 0.001^c^
**Myocardial injury markers**					
CK (U/L)	73 (51, 103)	74 (55, 107)	70 (49, 96)	92 (60, 159)	0.023^a^; 0.041^b^; 0.007^c^
CK-MB (U/L)	12 (10, 15)	13 (10, 15)	12 (10, 15)	13 (10, 16)	0.065; 0.392; 0.106
LDH (U/L)	193 (166, 223)	196 (171, 223)	187 (164, 220)	229 (189, 285)	0.029^a^; < 0.001^b^; < 0.001^c^
**Coagulative markers**					
PT (s)	11.2 (10.7, 11.8)	10.9 (10.5, 11.5)	11.3 (10.9, 11.9)	12.7 (11.7, 13.6)	< 0.001^a^; < 0.001^b^; < 0.001^c^
PTA (%)	97 (90, 105)	101 (93, 108)	95 (88, 101)	80 (72, 92)	< 0.001^a^; < 0.001^b^; < 0.001^c^
INR	1 (0.96, 1.05)	0.99 (0.93, 1.02)	1.00 (0.99, 1.07)	1.10 (1.01, 1.20)	< 0.001^a^; < 0.001^b^; < 0.001^c^
APTT (s)	30.5 (28.5, 33.1)	30.5 (28.7, 33.1)	30.7 (28.5, 33)	29.8 (27.4, 32.3)	0.868; 0.105; 0.11^c^
FIB (g/L)	3.03 (2.67, 3.48)	3.08 (2.71, 3.51)	2.96 (2.61, 3.37)	3.72 (3.12, 4.95)	< 0.001^a^; < 0.001^b^; < 0.001^c^
TT (s)	14.1 (13.3, 14.9)	13.7 (13.2, 14.6)	14.2 (13.5, 15.1)	14.1 (13.0, 15.2)	< 0.001^a^; 0.231; 0.47
DD (μg/mL)	0.41 (0.23, 0.78)	0.34 (0.20, 0.60)	0.44 (0.25, 0.84)	1.44 (0.82, 3.72)	< 0.001^a^; < 0.001^b^; < 0.001^c^
FDP (μg/mL)	1.04 (0.63, 2.00)	0.90 (0.59, 1.44)	1.12 (0.66, 2.16)	2.93 (1.81, 7.65)	< 0.001^a^; < 0.001^b^; < 0.001^c^

AIS, acute ischemic stroke; TOAST, Trial of Org 10172 in Acute Stroke Treatment; LAA, large-artery atherosclerosis; SAO, small-artery occlusion; NIHSS, the National Institutes of Health Stroke Scale; GCS, Glasgow coma scale; mRS, modified Rankin scale; HTN, hypertension; AF, atrial fibrillation; CHD, coronary heart disease; DM, diabetes mellitus; IMT, intima-media thickness; CP, carotid plaque; VP, vulnerable plaque; SP, stable plaque; CS, carotid stenosis; HR, heart rate; SaO_2_, oxygen saturation in arterial blood; SBP, systolic blood pressure; DBP, diastolic blood pressures; BMI, body mass index; WBC, white blood cell; NEU, neutrophil; LYM, lymphocyte; MON, monocyte; NLR, neutrophil to lymphocyte ratio; LMR, lymphocyte to monocyte ratio; MHR, monocyte to high-density lipoprotein-cholesterol ratio; NHR, neutrophil to high-density lipoprotein-cholesterol ratio; SII, systemic immune-inflammation index; SIRI, system inflammation response index; MII-1, multi-inflammatory index-1; MII-2, multi-inflammatory index-2; MII-3, multi-inflammatory index-3; RPR, red blood cell distribution width to platelet ratio; CRP, C-reaction protein; RBC, red blood cell; HGB, hemoglobin; HCT, hematocrit; MCV, mean corpuscular volume; MCH, mean corpuscular hemoglobin; MCHC, mean corpuscular hemoglobin concentration; RDWSD, red blood cell distribution width standard deviation; RDWCV, red blood cell distribution width coefficient of variation; TC, total cholesterol; TG, total triglyceride; HDL-C, high-density lipoprotein-cholesterol; LDL-C, low-density lipoprotein cholesterol; AIP, atherogenic index of plasma; LCI, lipoprotein combine index; AC, atherogenic coefficient; CRI-I, Castelli's index-I; CRI-II, Castelli's index-II; non-HDL, non-high density lipoprotein-cholesterol; GLU, glucose; TyG, triglyceride-glucose; CREA, creatinine; UCR, urea to creatinine ratio; UA, uric acid; K, potassium; Na, sodium; Cl, chlorine; CO_2_, carbon dioxide; Ca, calcium; P, phosphorus; Mg, magnesium; TBIL, total bilirubin; DBIL, direct bilirubin; IBIL, indirect bilirubin; ALT, alanine transaminase; AST, aspartate aminotransferase; AAR, aspartate aminotransferase to alanine transaminase ratio; GGT, γ glutamyl transpeptadase; ALP, alkaline phosphatase; CHE, cholinesterase; TP, total protein; ALB, albumin; GLB, globulin; AGR, albumin to globulin ratio; CK, creatine kinase; CK-MB, creatine kinase-MB; LDH, lactic dehydrogenase; PT, prothrombin time; PTA, prothrombin activity; INR, international normalized ratio; APTT, activated partial thromboplastin time; FIB, fibrinogen; TT, thrombin time; FDP, fibrin degradation products; DD, D-Dimer. ^a^Comparison between phenotype 1 and phenotype 2 with p < 0.05; ^b^comparison between phenotype 1 and phenotype 3 with p < 0.05; ^c^comparison between phenotype 2 and phenotype 3 with p < 0.05.

In phenotype 1, the lipid parameters, including TC, TG, LDL-C, AIP, LCI, non-HDL-C, AC, CRI-I, and CRI-II, were significantly higher than the other two phenotypes (all *p* < 0.05). In phenotype 2, elevated levels of sodium (Na) and chloride (Cl) ions were found, compared to phenotype 1 and 3 (all *p* < 0.05). Nevertheless, patients in phenotype 3 had significant inflammation levels. They had abnormally increasing white blood cell (WBC), NEU, MON, NLR, MHR, NHR, SII, SIRI, MII-1, MII-2, MII-3, CRP, and lower levels of LYM and LMR inflammatory indicators, among the three phenotypes (all *p* < 0.05). Besides, phenotype 3 also had mild multiple-organ dysfunction, such as abnormal synthesis, secretion, coagulation, and excretion function occurring in the liver and renal, as well as myocardial injury. The basic characteristics of phenotypes and non-AIS control groups are displayed in Supplementary Table 1.

In the external validation dataset, 507 AIS patients were also divided into three clusters by the k-means cluster algorithm (Figures 2C, D), including clusters A (*n* = 251), B (*n* = 213), and C (*n* = 43). We compared the differences between the three groups in terms of clinical and laboratory data. Cluster A was characterized by abnormal ions, especially Na and Cl ions, corresponding to phenotype 2. Cluster B had high levels of lipid and BMI, which was equal to phenotype 1. Cluster C had mild organ dysfunction and severe levels of inflammation, with abnormal elevated and decreased inflammatory indicators, similar to phenotype 3. Supplementary Table 2 describes the detailed results.

### Clinlabomics models of phenotypes

**Figure 4:**
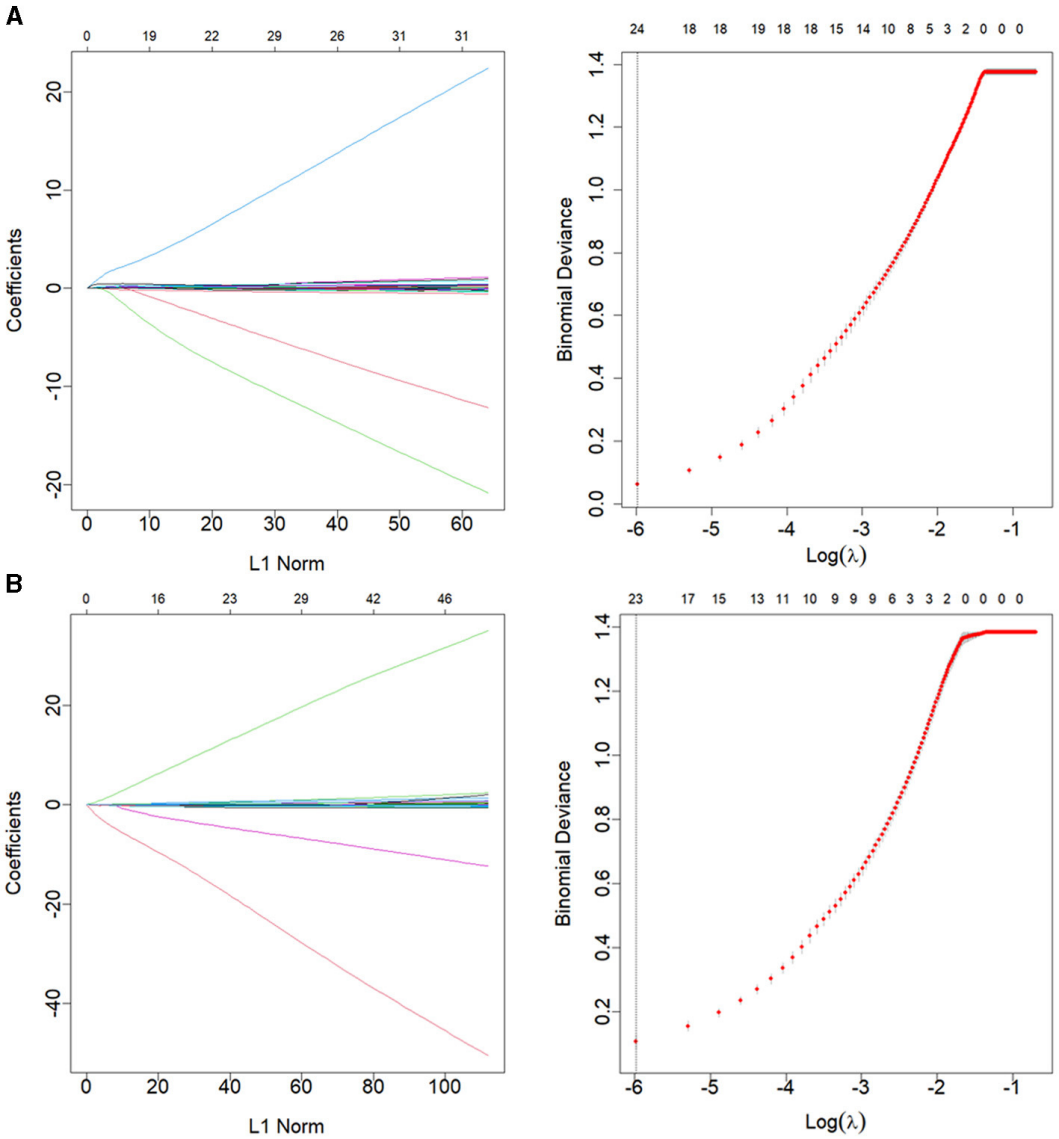
LASSO regression analysis for variable selection of **(A)** phenotype 1 and **(B)** phenotype 2. The LASSO coefficient profiles (left) and selection of the λ by 10-fold cross-validation in the LASSO analysis (right). LASSO, least absolute shrinkage, and selection operator.

**Figure 5:**
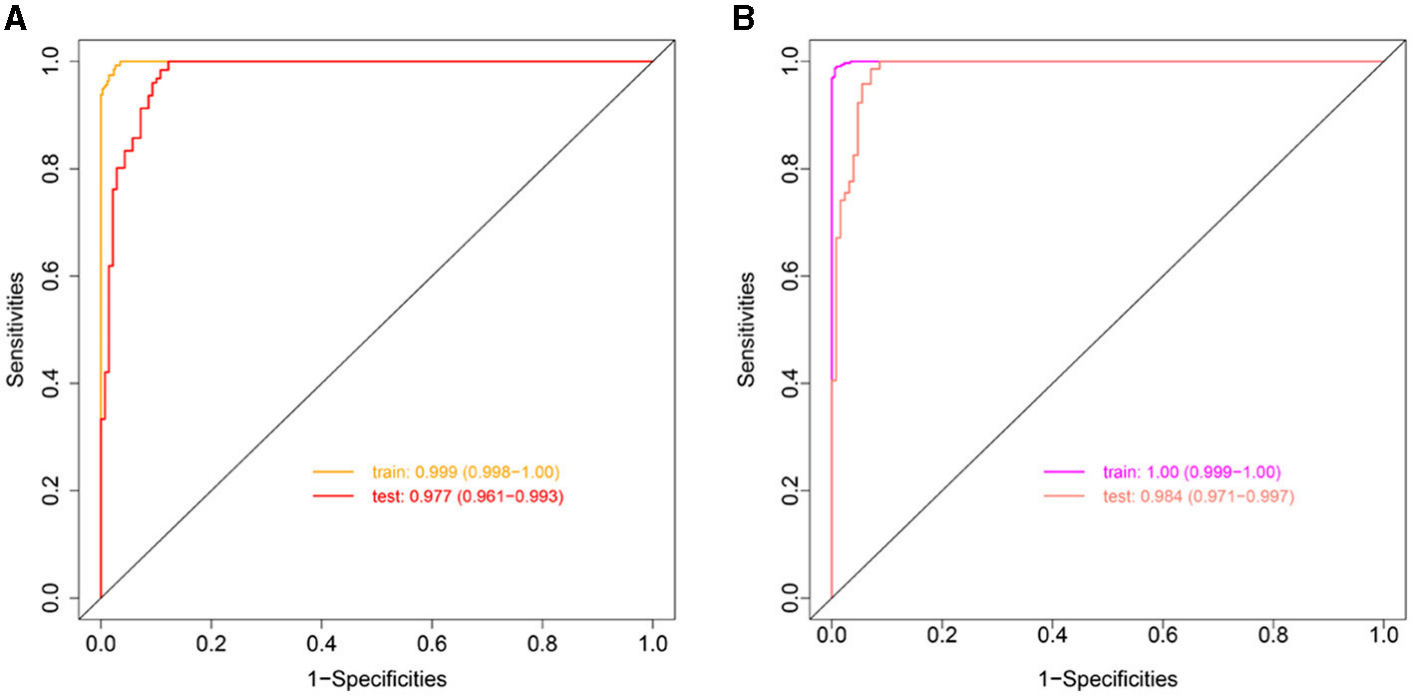
ROC curves of Clinlabomics **(A)** model 1 and **(B)** model 2. ROC, receiver-operating characteristic; AUC, the area under curve.

**Figure 6:**
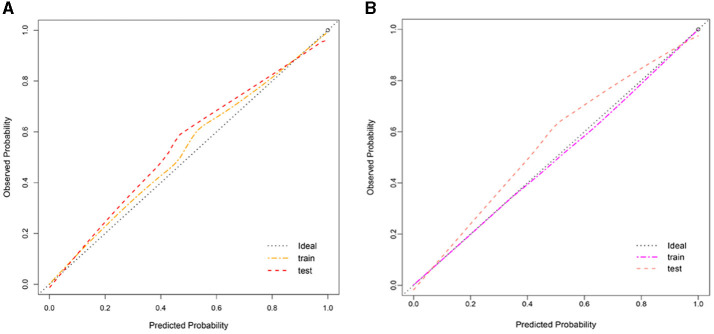
Calibration plot of Clinlabomics **(A)** model 1 and **(B)** model 2.

**Figure 7:**
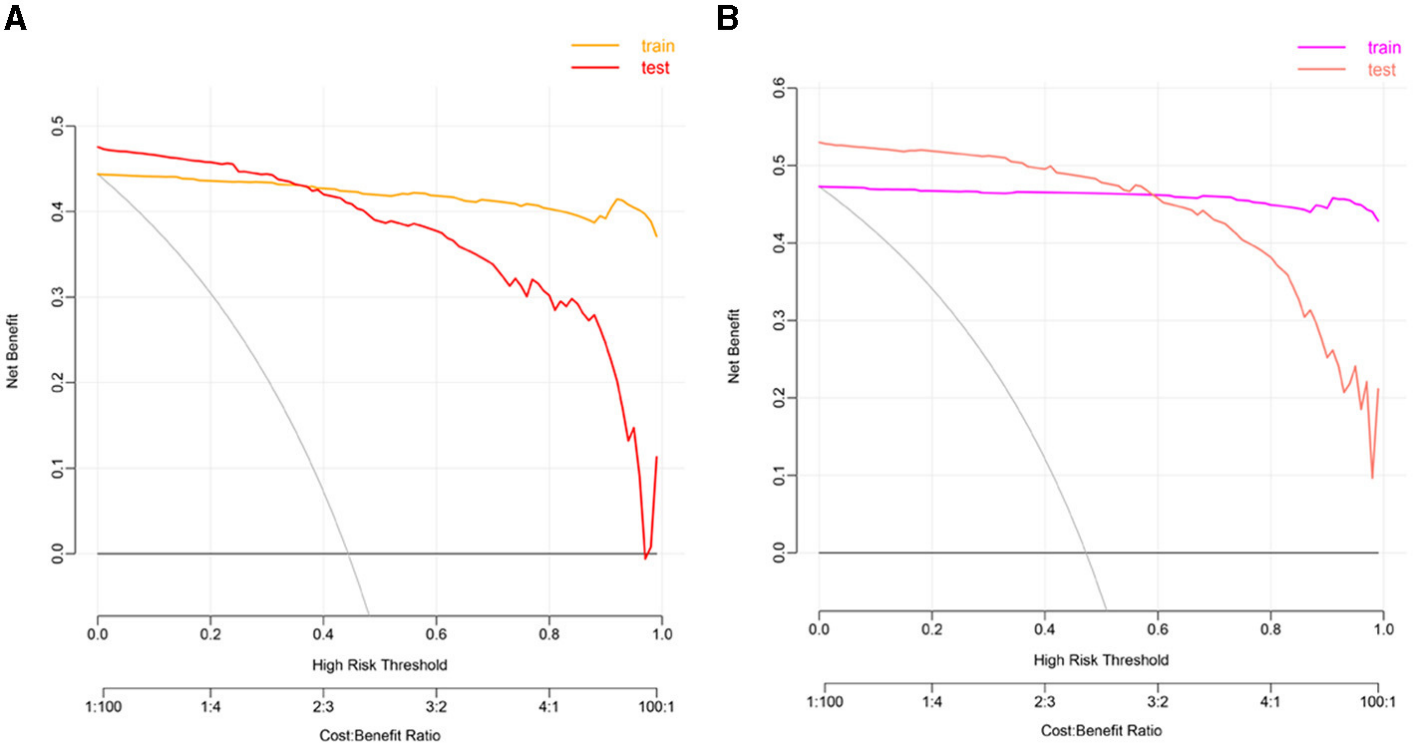
The DCA plots of Clinlabomics **(A)** model 1 and **(B)** model 2. DCA, decision curve analysis.

We used LASSO regression analysis to select 24 variables for the establishment of Clinlabomics model 1 of phenotype 1, including age, hypertension (HTN), smoking, systolic blood pressure (SBP), WBC, LYM, SII, MII-2, RPR, CRP, RBC, mean corpuscular volume (MCV), RDWCV, LDL-C, CRI-II, glucose (GLU), TyG, Cl, Ca, direct bilirubin (DBIL), alkaline phosphatase (ALP), cholinesterase (CHE), AGR, and PT (Figure 4A). For constructing predictive model 2 of phenotype 2 (Figure 4B), 23 variables, namely age, marriage, CHD, AF, drinking, HR, SBP, weight, LYM, NHR, SII, MII-2, RPR, CRP, TG, LCI, GLU, carbon dioxide (CO_2_), magnesium (Mg), ALB, AGR, INR, and TT were identified using a LASSO method. The predictive performance of the two phenotype classifiers established by the SVM algorithm was excellent, achieving high AUC values (ranging from 0.961 to 1.00), as shown in Figure 5 and Table 3. In particular, model 2 yielded higher accuracy (0.991 and 0.952), sensitivity (0.991 and 0.958), specificity (0.992 and 0.945), PPV (0.991 and 0.951), and NPV (0.992 and 0.952) both in training and testing datasets. Additionally, we selected a relatively important ranking of the top ten variables of models (Supplementary Figure 1). Notably, the inflammatory biomarkers CRP, RPR, and MII-2 were extremely important variables that ranked in the top three, both in model 1 and model 2. Furthermore, the calibration plots of the models showed a good agreement between the predicted probability and observed probability (Figure 6). Decision curve analysis (DCA) curves of two phenotype classifiers denoted optimal clinical efficacy (Figure 7).

**Table 3:** Evaluation metrics assess the predictive performance of Clinlabomics models.

**Evaluation metrics**	**Model 1 (95% CI)**		**Model 2 (95% CI)**	
	**Training**	**Testing**	**Training**	**Testing**
AUC (95% CI)	0.999 (0.998–1.00)	0.977 (0.961–0.993)	1.00 (0.999–1.00)	0.984 (0.971–0.997)
ACC	0.982 (0.975–0.991)	0.936 (0.922–0.956)	0.991 (0.986- 0.994)	0.952 (0.938–0.972)
Sensitivity	0.993 (0.981–0.996)	0.984 (0.968–0.998)	0.991 (0.985–0.997)	0.958 (0.939–0.984)
Specificity	0.974 (0.961–0.990)	0.892 (0.874–0.926)	0.992 (0.984–0.999)	0.945 (0.923–0.969)
PPV	0.968 (0.953–0.988)	0.892 (0.875–0.923)	0.991 (0.982–0.999)	0.951 (0.935–0.972)
NPV	0.994 (0.985–0.997)	0.984 (0.969–0.998)	0.992 (0.987–0.997)	0.952 (0.932–0.982)
Threshold	0.428	-	0.547	-
Youden	1.967	1.876	1.982	1.903

CI, confidence interval; AUC, the area under curve; ACC, accuracy; PPV, positive predictive value; NPV, negative predictive value; -, not available.

## Discussion

In this retrospective analysis of data from AIS patients, we classified them into three novel phenotypes with distinct clinical characteristics and significantly different laboratory data. This stratification of AIS patients may provide evidence of potential pathophysiology mechanisms of diseases and can help clinicians make clinical decisions about the intervention of stroke.

Of the three novel phenotypes, phenotype 3, which had only ~5% of the overall population sample size, was closely related to the older adult population and had the highest level of inflammation and mild multiple-organ dysfunction, containing abnormal liver, kidney function, and coagulative status. While phenotype 2 was characterized by a mild increase in inflammatory markers, it had the lowest lipid levels. Interestingly, the serum ions, such as potassium (K), NA, Cl, CO_2_, and phosphorus (P), were observed to be increased in phenotype 2. In contrast, phenotype 1 had a relatively young but high BMI population, who had significantly elevated levels of lipids.

We also compared with other phenotypes of ischemic stroke (Table 4). For instance, Chen and Chen (8) and Lattanzi et al. (10) revealed a clinical phenotype with dyslipidemia in embolic stroke of undetermined source (ESUS) and ischemic stroke with OSA, respectively. Likewise, Ding et al. (6, 7) also identified the phenotypes of abnormal inflammation and lipid metabolism of NCIS patients, which demonstrated that inflammatory and lipid alterations were closely associated with the occurrence of ischemic stroke. In our study, we found a distinct phenotype with abnormal ions for the first time, which may provide new insight into targeted treatments of AIS patients.

**Table 4:** Comparison with other phenotypes of ischemic stroke.

**Author**	**Year**	**No. patients**	**The source of patients**	**Diseases**	**Methods**	**Variables**	**No. of phenotypes**	**Traits of phenotypes**
Chen and Chen (8)	2021	232	Chang Gung Memorial Hospital	Ischemic stroke with OSA	LCA	13 variables: sex, age, smoking, daytime sleepiness, depression, obesity, sedative use, AF, DM, HTN, dyslipidemia, recurrent stroke, and dysphagia	Three	Cluster 1 (*N =* 84): older, predominantly female, the highest hypopnea index and prevalence of AF. Cluster 2 (*N =* 80): older, predominantly male, with the highest depression, the lowest prevalence of HTN, and normal BMI. Cluster 3 (*N =* 68): the youngest, predominantly male, with the highest BMI, cumulative risk score, and prevalence of dyslipidemia.
Ding et al. (7)	2023	7695	CNSR-III	NCIS	Ward's hierarchical agglomerative clustering method	63 biomarkers: ANGPTL3, PCSK9, Lp-PLA2-Activity, LDL, Lp, ADPN, HDL, LDL-R, TG, ApoE, ApoAI, ApoAII, ApoB, MCV, B, E, PLT, RDWCV, MPV, HGB, MCHC, APTT, INR, PT, FIB, D-D, TT, Cl, Na, K, FPG, TMAVA, TMAO, TML, Carnitine, Butyrobetaine, Betaine, Choline, MMA, HCY, Folic acid, Vitamin B12, MON, NEU, LYM, IL-6, hs-CRP, IL-1Ra, YKL-40, MCP-1, IL-6R, ALP, GLB, GGT, ALT, AST, DBIL, IBIL, ALB, UA, CysC, CREA, and UMA	30	C1 (*N =* 53): hs-CRP, history of stroke. C2 (*N =* 70): D-dimer. C3 (*N =* 194): MON, NEU. C4 (*N =* 308): IL-6, age (median 67, IQR: 60−76) C5 (*N =* 49): UMA, history of stroke, T2DM HTN, family history of DM, HTN, and stroke. C6 (*N =* 88): TMAO, liver disease. C7 (*N =* 153): CysC, CREA, age (median 68, IQR 61 - 76), history of stroke, HTN, CHD. C8 (*N =* 81): MMA, family history of HTN. C9 (*N =* 183): HCY, smoking. C10 (*N =* 211): Folic acid. C11 (*N =* 677): APTT, INR, PT. C12 (*N =* 991): ADPN, HDL. C13 (*N =* 569): ADPN, HDL, YKL-40, BMI (median 23.67, IQR: 21.78 - 25.53). C14 (*N =* 125): TML, carnitine, butyrobetaine, betaine, choline. C15 (*N =* 101): RDWCV, MCV, HGB, MCHC. C16 (*N =* 128): ApoAI. C17 (*N =* 128): ALT, AST, liver disease. C18 (*N =* 158): GGT, smoking, drinking, liver disease. C19 (*N =* 178): LDL-R, Apo-E, TG, hyperlipemia. C20 (*N =* 89): MCP-1. C21 (*N =* 135): IL-1Ra. C22 (*N =* 264): Vitamin B12, PAD. C23 (*N =* 359): B, E, smoking. C24 (*N =* 827): TMAVA, TML. C25 (*N =* 114): Lp (a). C26 (*N =* 149): LDL, hyperlipemia. C27 (*N =* 144): DBIL, IBIL. C28 (*N =* 707): BMI: 25.39 (23.56, 27.60). C29 (*N =* 214): FPG, T2DM, family history of DM. C30 (*N =* 248): Apo-AII, Apo-B.
Ding et al. (6)	2022	9288	CNSR-III	NCIS	GMM clustering method	30 features: BMI, SBP, DBP, MMA, HCT, MCV, PLT, RBC, APTT, TT, K, CREA, UA, MON, NEU, LYM, hs-CRP, FPG, HDL, TC, LDL, Lp (a), TG, GGT, DBIL, TP, ALP, ALT, Choline, and infarct volume.	Four	Phenotype 1: abnormal glucose and lipid metabolism. Phenotype 2: inflammation and abnormal renal function. Phenotype 3: the least laboratory abnormalities and small infarct lesions. Phenotype 4: disturbance in homocysteine metabolism.
Lattanzi et al. (10)	2021	127	The Marche Polytechnic University	ESUS	HCA	Two variables: age and baseline NIHSS	Three	Cluster 1: young age, male sex, posterior circulation infarct, and presence of PFO. Cluster 2: HTN, DM, severe stroke, involvement of multiple vascular territories, and left atrial cardiopathy. Cluster 3: dyslipidemia, smoking, infarct of anterior vascular territory, and ipsilateral non-stenotic vulnerable carotid plaque.
Schütz et al. (9)	2019	451	Nueces County, Texas, residents	Ischemic stroke with OSA	LCA	15 variables: snoring, tiredness/fatigue, history of prior stroke/TIA, congestive heart failure, CAD, DM, HTN, sex, race/ethnicity, AF, sleep duration, age, BMI, NIHSS, and REI.	Three	Cluster 1: Severe strokes. Cluster 2: Younger patients with mild strokes and relatively mild OSA. Cluster 3: Severe OSA with high prevalence of co-morbidities.
This study	2024	909	Lanzhou University Second Hospital	AIS	k-means clustering method	76 variables: HR, SBP, DBP, SaO_2_, weight, height, BMI, WBC, NEU, LYM, MON, NLR, LMR, MHR, NHR, SII, SIRI, MII-1, MII-2, MII-3, RPR, CRP, RBC, HGB, HCT, MCV, MCH, MCHC, RDWCV, TC, TG, HDL-C, LDL-C, AIP, LCI, non-HDL-C, AC, CRI-I, CRI-II, GLU, TyG, UREA, CREA, UCR, UA, K, NA, Cl, CO_2_, Ca, P, Mg, TBIL, DBIL, IBIL, ALT, AST, AAR, GGT, ALP, CHE, TP, ALB, GLB, AGR, CK, CK-MB, LDH, PT, PTA, INR, APTT, FIB, TT, DD, and FDP	Three	Phenotype 1: relatively young and obese and significantly elevated levels of lipids. Phenotype 2: abnormal ion levels. Phenotype 3: the highest level of inflammation, mild multiple-organ dysfunction.

OSA, obstructive sleep apnea; LCA, latent class analysis; AF, atrial fibrillation; DM, diabetes mellitus; HTN, hypertension; BMI, body mass index; CNSR-III, Third China National Stroke Registry; NCIS, Non-cardioembolic ischemic stroke; ANGPTL3, Angiopoietin-Like 3; PCSK9, proprotein convertase subtilisin/kexin type 9; Lp-PLA2, lipoprotein-associated phospholipase 2; LDL, low-density lipoprotein cholesterol; Lp, lipoprotein; ADPN, adiponectin; HDL, high-density lipoprotein; LDL-R, low-density lipoprotein receptor; TG, total triglyceride; ApoE, apolipoprotein E; ApoAI, apolipoprotein AI; ApoAII, apolipoprotein AII; ApoB, apolipoprotein B; MCV, mean corpuscular volume; B, Basophil; E, Eosinophil; PLT, platelet; RDWCV, red blood cell distribution width coefficient of variation; MPV, mean platelet volume; HGB, hemoglobin; MCHC, mean corpuscular hemoglobin concentration; APTT, activated partial thromboplastin time; INR, international normalized ratio; PT, prothrombin time; FIB, fibrinogen; D-D, D-Dimer; TT, thrombin time; Cl, chlorine; Na, sodium; K, potassium; FPG, fasting plasma glucose; TMAVA, N,N,N-trimethyl-5-aminovaleric acid; TMAO, trimethylamine-N-oxide; TML, trimethyllysine; MMA, methylmalonic aciduria; HCY, homocysteine; MON, monocyte; NEU, neutrophil; LYM, lymphocyte; IL-6, interleukin- 6; hs-CRP, hypersensitive C-reactive protein; IL-1Ra, Interleukin-1 receptor antagonist; YKL-40, chitinase-3-like protein 1; MCP-1, monocyte chemoattractant protein-1; IL-6R, interleukin-6 receptor; ALP, alkaline phosphatase; GLB, globulin; GGT, γ glutamyl transpeptadase; ALT, alanine transaminase; AST, aspartate aminotransferase; DBIL, direct bilirubin; IBIL, indirect bilirubin; ALB, albumin; UA, uric acid; CysC, Cystatin C; CREA, creatinine; UMA; renal function index; GMM, Gaussian mixture model; SBP, systolic blood pressure; DBP, diastolic blood pressures; MMA, methylmalonic aciduria; HCT, hematocrit; RBC, red blood cell; TC, total cholesterol; LDL, low-density lipoprotein; TP, total protein; ESUS, embolic stroke of undetermined source; HCA, hierarchical cluster analysis; NIHSS, the National Institutes of Health Stroke Scale; TIA, transient ischemic attack; CAD, coronary artery disease; REI, respiratory-event-index; AIS, acute ischemic stroke; HR, heart rate; SaO_2_, oxygen saturation in arterial blood; WBC, white blood cell; NLR, neutrophil to lymphocyte ratio; LMR, lymphocyte to monocyte ratio; MHR, monocyte to high-density lipoprotein-cholesterol ratio; NHR, neutrophil to high-density lipoprotein-cholesterol ratio; SII, systemic immune-inflammation inde; SIRI, system inflammation response index; MII-1, multi-inflammatory index-1; MII-2, multi-inflammatory index-2; MII-3, multi-inflammatory index-3; RPR, red blood cell distribution width to platelet ratio; CRP, C-reaction protein; MCH, mean corpuscular hemoglobin; AIP, atherogenic index of plasma; LCI, lipoprotein combine index; AC, atherogenic coefficient; CRI-I, Castelli's index-I; CRI-II, Castelli's index-II; non-HDL, non-high density lipoprotein-cholesterol; GLU, glucose; TyG, triglyceride-glucose; UCR, urea to creatinine ratio; CO_2_, carbon dioxide; Ca, calcium; P, phosphorus; Mg, magnesium; TBIL, total bilirubin; AAR, aspartate aminotransferase to alanine transaminase ratio; CHE, cholinesterase; AGR, albumin to globulin ratio; CK, creatine kinase; CK-MB, creatine kinase-MB; LDH, lactic dehydrogenase; PTA, prothrombin activity; FDP, fibrin degradation products.

Recent works have shown that inflammation plays a vital role in the pathogenesis of AIS, which may increase the risk of stroke and exacerbate ischemic lesions (37–39). When ischemia occurs in the cerebrum, peripheral circulating leukocytes and their subsets, including neutrophils, monocytes, and lymphocytes, are recruited to the cerebral ischemic region. These cells produce, secrete, and activate inflammatory mediators, such as cytokines, chemokines, adhesion molecules, etc., and even interact with inflammatory cells to contribute to the progression and sustenance of inflammation (40, 41). Inflammatory responses participate in the process of thrombosis, which, in turn, can generate a thrombotic inflammatory response via the recruitment of leukocytes, leading to tissue organ damage and influencing the clinical outcome of AIS patients (42). One collaborative analysis of 31,245 patients who received statin therapy revealed that residual inflammatory risk (RIR), namely LDL-C <70 mg/dL and high-sensitivity C-reactive protein (hs-CRP) level ≥ 2 mg/L, can effectively predict cardiovascular events and death, and all-cause death (43). Similarly, RIR was strongly associated with the poor functional outcome of AIS patients and could predict the risk of recurrent stroke for AIS or TIA patients (44). Therefore, an anti-inflammatory strategy is recognized as a potential treatment to reduce the recurrence of stroke and other vascular events after the onset of IS (45, 46).

Furthermore, we found that the levels of traditional lipid parameters, including TC, TG, HDL-C, and LDL-C, and non-traditional lipid parameters, such as AIP, LCI, non-HDL-C, AC, CRI-I, and CRI-II, were significantly increased in the phenotype 1, which had 46% carotid plaque occurrence rate in all AIS population. Abnormal lipid metabolism and inflammatory responses are involved in the pathological progression of atherosclerosis, which is initiated by oxidation of LDL-C, activated by endothelium, and mediated by macrophages (47). Hyperlipidemia can recruit pro-inflammatory monocytes, which infiltrate into atherosclerotic lesions and ultimately form foam cells. They also can activate the innate immune response by triggering the production of many pro-inflammatory cytokines. Importantly, inflammation and hyperlipidemia had similar future atherothrombotic risks in the population without receiving statins (43). Thus, it is important to understand the vital roles of inflammation and lipids in the atherosclerosis process for better intervention of IS. Currently, statin therapy is recommended to reduce cardiovascular event risk among people with atherosclerosis in primary or secondary prevention, based on the randomized trials that demonstrated the efficacy of statin to decline the occurrence of cardiovascular events in patients with high levels of LDL-C (48) and hs-CRP (49). In addition, other lipid-lowering therapies, including ezetimibe, bempedoic acid, proprotein convertase subtilisin-kexin type 9 (PCSK9) inhibitors, angiopoietin-like 3 protein (ANGPTL3) inhibitors, and inclisiran were also observed to reduce cardiovascular event rates (50–52). A parallel-group trial elucidated that a target LD-L cholesterol <70 mg/dL in IS or TIA patients with atherosclerosis had lower cardiovascular risk (53).

Interestingly, both inflammation biomarkers and lipid levels were found to be the lowest in phenotype 2, but the levels of K, NA, Cl, and P ions were increased. After the onset of cerebral ischemia, endogenous Na+/K+-ATPase (NKA) inhibitors that damaged the innate NKA activity were released to the peripheral circulation (54), leading to ATP depletion, which in turn exacerbated anoxic damage (55, 56). Besides, abnormal metabolic changes occurred in extracellular and intracellular environments, namely, reductions in ATP and cytosolic K^+^, as well as increases in ROS produced by mitochondria and intracellular Ca^2+^. These changes activated the nucleotide-binding oligomerization domain (NOD)-like receptor (NLR) family pyrin domain-containing 3 (NLRP3) inflammasome and subsequent pro-caspase-1 self-cleaved into caspase-1, mediating pyroptosis and ultimately causing neuronal death (57). In addition, the decline of intracellular K+ could also stimulate the activation of NLRP3 inflammasome and trigger inflammation cascades (58). Therefore, restoring the activity of NKA may reduce inflammasome activation, relieve neuronal death, and attenuate ischemic injury (59), which may be a distinct therapeutic target for AIS.

In this study, a total of 24 and 23 variables were selected to construct Clinlabomics models of phenotype 1 and phenotype 2, respectively. The SVM generally presented a similar or superior ability to the logistic regression (LR) method in the classification of diseases (60). We tried to use the LR algorithm to construct the Clinlabomics models of phenotypes, but the results were disappointing with the fitted probabilities numerically 0 or 1. Thus, we established the phenotype classifiers using the SVM algorithm, which showed excellent predictive performance for phenotypes of AIS patients. Both in models 1 and 2, CRP, RPR, and MII-2 inflammatory biomarkers were the most important predictors. Kitagawa et al. (61) revealed that a low level of CRP (<1 mg/L) reduced 32% recurrent stroke and TIA compared to patients with CRP ≥ 1 mg/L. In addition, elevated CRP was observed to be strongly correlated to a 3-month worse outcome of stroke patients without infection (62). The RPR, as a new inflammatory index, was closely related to the risk of mortality among AIS patients (63, 64). Furthermore, an increase in RPR could also predict early neurological deterioration after intravenous thrombolysis in patients with AIS (65). It remains unclear whether any relationship exists between the MII-2 indicator and AIS patients, but a recent study elucidated that the MII-1 and MII-2 inflammatory markers were capable of predicting the occurrence of acute symptomatic seizures after IS (66). With advances in algorithms to develop prediction models by combining multiple variables, we can optimize models to identify the hidden complex relationships among variables, which may be of great utility in clinical practice.

However, we should consider limitations on the interpretation of our findings. First, this is a single-center, small sample-size study that needs further validation in a large-scale study. Second, we also need to investigate more advanced ML algorithms to better predict the phenotypes of AIS patients based on multicenter and large-scale research. Third, due to the small population (*n* = 45), we did not establish the predictive phenotype classifiers of phenotype 3, which is required to explore the underlying mechanism of mild organ damage and dysfunction in the future. Interestingly, although a large quantity of ML-based models exists to predict AIS, they are not effectively utilized in clinical practice, which is ascribed to the complicated data mining algorithms and abstruse formulas. Therefore, it is imperative to solve this problem to better apply these models by clinicians.

## Conclusion

In conclusion, we identified three novel phenotypes that connected with different clinical variables using k-means clustering analysis. We constructed the Clinlabomics models of phenotypes in AIS patients that are conducive to clinical decision-making and personalized medicine.

## Data availability statement

The raw data supporting the conclusions of this article will be made available by the authors, without undue reservation.

## Ethics statement

The studies involving humans were approved by the Ethics Committee of the Lanzhou University Second Hospital (IRB number: 2022A-710). The studies were conducted in accordance with the local legislation and institutional requirements. The participants provided their written informed consent to participate in this study.

## Author contributions

YJ: Data curation, Formal analysis, Methodology, Writing—original draft, Visualization, Writing—review & editing. YD: Formal analysis, Investigation, Methodology, Validation, Visualization, Writing—original draft. QW: Methodology, Validation, Visualization, Writing—review & editing. BY: Software, Supervision, Writing—review & editing. LG: Funding acquisition, Writing—review & editing. CY: Conceptualization, Writing—review & editing.
