# Supplementary material for: Using a k-means clustering to identify novel phenotypes of acute ischemic stroke and development of its Clinlabomics models
Source: Front Neurol. 2024 Mar 27;15:1366307. doi: 10.3389/fneur.2024.1366307 (PMC11004235; doi:10.3389/fneur.2024.1366307)
Supplement: Supplementary file 1 [file Table_1.docx]

Supplementary Table 1 Comparison of the basic characteristics between phenotypes and non-AIS control groups

| Variables | Phenotype 1  (n = 401) | | Phenotype 2  (n = 463) | | Phenotype 3  (n = 45) | | | Non-AIS group  (n=484) | | Comparison *P* value between | | | |
| --- | --- | --- | --- | --- | --- | --- | --- | --- | --- | --- | --- | --- | --- |
| **Demographic characteristics** | | |  | |  | | |  | | Phenotype 1 and Control | Phenotype 2 and Control | Phenotype 3 and Control | |
| Age | 61 (53, 70) | | 66 (56, 74) | | 70 (54, 75) | | | 66 (57, 72) | | < 0.001 | 0.244 | | 0.275 |
| Gender |  | |  | |  | | |  | |  |  | |  |
| Female (%) | 120 (30) | | 147 (32) | | 14 (31) | | | 226 (47) | | <0.001 | <0.001 | | 0.064 |
| Male (%) | 281 (70) | | 316 (68) | | 31 (69) | | | 258 (53) | |  |  | |  |
| Nationality |  | |  | |  | | |  | |  |  | |  |
| Han (%) | 377 (94) | | 426 (92) | | 42 (93) | | | 449 (93) | | 0.545 | 0.75 | | 1 |
| Minority (%) | 24 (6) | | 37 (8) | | 3 (7) | | | 35 (7) | |  |  | |  |
| Marriage |  | |  | |  | | |  | |  |  | |  |
| Married (%) | 391 (98) | | 457 (99) | | 45 (100) | | | 481 (99) | | 0.043 | 0.332 | | 1 |
| Other status (%) | 10 (2) | | 6 (1) | | 0 (0) | | | 3 (1) | |  |  | |  |
| Education |  | |  | |  | | |  | |  |  | |  |
| High school diploma or higher (%) | 122 (30) | | 151 (33) | | 12 (27) | | | 188 (39) | | 0.011 | 0.053 | | 0.147 |
| Others (%) | 279 (70) | | 312 (67) | | 33 (73) | | | 296 (61) | |  |  | |  |
| **Previous history** | | |  | |  | | |  | |  |  | |  |
| HTN |  | |  | |  | | |  | |  |  | |  |
| No (%) | 162 (40) | | 213 (46) | | 23 (51) | | | 267 (55) | | < 0.001 | 0.006 | | < 0.001 |
| Yes (%) | 239 (60) | | 250 (54) | | 22 (49) | | | 217 (45) | |  |  | |  |
| AF |  | |  | |  | | |  | |  |  | |  |
| No (%) | 396 (99) | | 453 (98) | | 43 (96) | | | 481 (99) | | 0.479 | 0.079 | | 0.479 |
| Yes (%) | 5 (1) | | 10 (2) | | 2 (4) | | | 3 (1) | |  |  | |  |
| CHD |  | |  | |  | | |  | |  |  | |  |
| No (%) | 390 (97) | | 440 (95) | | 43 (96) | | | 457 (94) | | 0.057 | 0.783 | | 0.057 |
| Yes (%) | 11 (3) | | 23 (5) | | 2 (4) | | | 27 (6) | |  |  | |  |
| DM |  | |  | |  | | |  | |  |  | |  |
| No (%) | 298 (74) | | 384 (83) | | 38 (84) | | | 417 (86) | | < 0.001 | 0.2 | | < 0.001 |
| Yes (%) | 103 (26) | | 79 (17) | | 7 (16) | | | 67 (14) | |  |  | |  |
| **Unhealthy habits** | | |  | |  | | |  | |  |  | |  |
| Smoking |  | |  | |  | | |  | |  |  | |  |
| No (%) | 308 (77) | | 368 (79) | | 30 (67) | | | 434 (90) | | < 0.001 | < 0.001 | | < 0.001 |
| Yes (%) | 93 (23) | | 95 (21) | | 15 (33) | | | 50 (10) | |  |  | |  |
| Drinking |  | |  | |  | | |  | |  |  | |  |
| No (%) | 365 (91) | | 430 (93) | | 37 (82) | | | 465 (96) | | 0.003 | 0.043 | | 0.003 |
| Yes (%) | 36 (9) | | 33 (7) | | 8 (18) | | | 19 (4) | |  |  | |  |
| **Non-invasive physiological indices** | | |  | |  | | |  | |  |  | |  |
| HR (bpm) | 78 (72, 88) | | 75 (68, 83) | | 86 (76, 100) | | | 77 (70, 85) | | 0.009 | 0.027 | | < 0.001 |
| SBP (mmHg) | 143 (130, 157) | | 138 (124, 152) | | 148 (140, 161) | | | 128 (119, 142) | | < 0.001 | < 0.001 | | < 0.001 |
| DBP (mmHg) | 85 (76, 93) | | 79 (71, 88) | | 80 (73, 93) | | | 76 (70, 84) | | < 0.001 | 0.001 | | 0.016 |
| SaO2 (%) | 96 (94, 96) | | 96 (94, 96) | | 96 (94, 98) | | | 95 (94, 96) | | 0.184 | 0.218 | | 0.535 |
| **Individual characteristics** | | |  | |  | | |  | |  |  | |  |
| Weight (Kg) | 70 (64, 75) | | 65 (60, 74) | | 65 (60, 70) | | | 65 (59, 74) | | < 0.001 | 0.559 | | 0.532 |
| Height (cm) | 168 (160, 172) | | 168 (160, 172) | | 170 (160, 174) | | | 165 (160, 170) | | < 0.001 | 0.001 | | 0.027 |
| BMI (Kg/m^2) | 25.06 (23.15, 27.34) | | 23.88 (22.04, 25.76) | | 22.86 (20.76, 24.8) | | | 24.16 (22.26, 26.08) | | < 0.001 | 0.129 | | 0.032 |
| **Inflammatory biomarkers** | | |  | |  | | |  | |  |  | |  |
| WBC (10^9^/L) | 6.94 (5.89, 8.46) | | 5.92 (4.98, 7.38) | | 12.00 (9.00, 15.32) | | | 5.60 (4.61, 6.66) | | < 0.001 | < 0.001 | | < 0.001 |
| NEU (10^9^/L) | 4.52 (3.54, 5.73) | | 3.76 (2.88, 4.95) | | 10.45 (7.27, 12.99) | | | 3.14 (2.46, 3.91) | | < 0.001 | < 0.001 | | < 0.001 |
| LYM (10^9^/L) | 1.75 (1.42, 2.21) | | 1.55 (1.17, 1.92) | | 0.86 (0.61, 1.29) | | | 1.70 (1.38, 2.10) | | 0.093 | < 0.001 | | < 0.001 |
| MON (10^9^/L) | 0.45 (0.36, 0.59) | | 0.45 (0.36, 0.56) | | 0.64 (0.51, 1.04) | | | 0.42 (0.33, 0.52) | | < 0.001 | 0.005 | | < 0.001 |
| NLR | 2.47 (1.84, 3.57) | | 2.45 (1.75, 3.5) | | 9.80 (6.82, 19.98) | | | 1.85 (1.38, 2.46) | | < 0.001 | < 0.001 | | < 0.001 |
| LMR | 3.90 (2.89, 5.07) | | 3.39 (2.59, 4.42) | | 1.40 (0.87, 1.74) | | | 4.05 (3.14, 5.14) | | 0.115 | < 0.001 | | < 0.001 |
| MHR | 0.44 (0.32, 0.61) | | 0.46 (0.34, 0.59) | | 0.79 (0.56, 1.08) | | | 0.38 (0.29, 0.53) | | < 0.001 | < 0.001 | | < 0.001 |
| NHR | 4.30 (3.34, 6.10) | | 3.81 (2.78, 5.30) | | 10.47 (7.76, 12.75) | | | 2.89 (2.14, 3.90) | | < 0.001 | < 0.001 | | < 0.001 |
| SII (10^9^/L) | 501 (341, 777) | | 439 (296, 678) | | 1945 (1222, 3016) | | | 330 (232, 472) | | < 0.001 | < 0.001 | | < 0.001 |
| SIRI (10^9^/L) | 1.12 (0.78, 1.80) | | 1.13 (0.74, 1.77) | | 8.57 (3.75, 16.43) | | | 0.79 (0.51, 1.13) | | < 0.001 | < 0.001 | | < 0.001 |
| MII-1 | 7.21 (2.73, 14.46) | | 7.67 (2.14, 14.99) | | 82.96 (24.98, 303.47) | | | 5.00 (2.07, 7.89) | | < 0.001 | < 0.001 | | < 0.001 |
| MII-2 | 325 (120, 679) | | 321 (98, 695) | | 1297 (301, 10385) | | | 115 (68, 283) | | < 0.001 | < 0.001 | | < 0.001 |
| MII-3 | 1509 (534, 2959) | | 1184 (339, 2906) | | 13549 (4224, 84269) | | | 841 (353, 1496) | | < 0.001 | < 0.001 | | < 0.001 |
| RPR | 0.06 (0.05, 0.07) | | 0.07 (0.06, 0.09) | | 0.08 (0.06, 0.11) | | | 3.70 (1.01, 3.70) | | < 0.001 | < 0.001 | | < 0.001 |
| CRP (mg/L) | 2.67 (1.09, 5.96) | | 2.79 (0.88, 5.96) | | 7.85 (2.14, 59.19) | | | 0.07 (0.06, 0.09) | | < 0.001 | < 0.001 | | < 0.001 |
| **Red blood cell related parameters** | | |  | |  | | |  | |  |  | |  |
| RBC (10^12^/L) | 4.96 (4.65, 5.32) | | 4.64 (4.25, 4.96) | | 4.33 (3.91, 4.99) | | | 4.59 (4.29, 4.97) | | < 0.001 | 0.758 | | 0.008 |
| HGB (g/L) | 155 (145, 164) | | 145 (135, 155) | | 136 (117, 153) | | | 143 (133, 156) | | < 0.001 | 0.597 | | 0.004 |
| HCT | 0.46 (0.43, 0.49) | | 0.43 (0.40, 0.46) | | 0.40 (0.35, 0.45) | | | 0.43 (0.40, 0.46) | | < 0.001 | 0.695 | | < 0.001 |
| MCV (fL) | 91.9 (89.1, 94.8) | | 93.6 (90.6, 96.9) | | 92.8 (89.0, 96.4) | | | 93.8 (90.9, 96.5) | | < 0.001 | 0.978 | | 0.154 |
| MCH (pg) | 31.0 (30.0, 32.1) | | 31.5 (30.3, 32.6) | | 31.4 (30.1, 32.8) | | | 31.3 (30.1, 32.4) | | 0.038 | 0.069 | | 0.784 |
| MCHC (g/L) | 337 (330, 343) | | 335 (328, 342) | | 337 (325, 348) | | | 332 (325, 340) | | < 0.001 | 0.001 | | 0.074 |
| RDWCV (%) | 12.6 (12.0, 13.2) | | 13.0 (12.4, 13.5) | | 13.5 (12.7, 14.0) | | | 12.9 (12.4, 13.3) | | < 0.001 | 0.05 | | < 0.001 |
| **Lipid parameters** | | |  | |  | | |  | |  |  | |  |
| TC (mmol/L) | 4.70 (4.12, 5.33) | | 3.43 (2.99, 4.00) | | 3.59 (2.95, 4.13) | | | 4.06 (3.36, 4.76) | | < 0.001 | < 0.001 | | 0.004 |
| TG (mmol/L) | 1.96 (1.55, 2.62) | | 1.13 (0.88, 1.40) | | 1.22 (0.80, 1.52) | | | 1.31 (0.96, 1.77) | | < 0.001 | < 0.001 | | 0.05 |
| HDL-C (mmol/L) | 1.03 (0.89, 1.21) | | 0.98 (0.85, 1.15) | | 0.98 (0.72, 1.21) | | | 1.10 (0.94, 1.27) | | < 0.001 | < 0.001 | | 0.007 |
| LDL-C (mmol/L) | 3.19 (2.76, 3.69) | | 2.29 (1.89, 2.67) | | 2.26 (1.73, 2.82) | | | 2.66 (2.12, 3.24) | | < 0.001 | < 0.001 | | 0.003 |
| AIP | 0.28 (0.14, 0.44) | | 0.06 (-0.08, 0.17) | | 0.06 (-0.06, 0.25) | | | 0.08 (-0.08, 0.24) | | < 0.001 | 0.049 | | 0.84 |
| LCI | 28.65 (19.13, 44.02) | | 8.93 (5.76, 13.34) | | 9.55 (4.96, 19.98) | | | 12.91 (7.33, 21.99) | | < 0.001 | < 0.001 | | 0.074 |
| non-HDL-C (mmol/L) | 3.64 (3.12, 4.19) | | 2.46 (2.04, 2.95) | | 2.56 (1.95, 3.14) | | | 2.91 (2.32, 3.58) | | < 0.001 | < 0.001 | | 0.025 |
| AC | 3.56 (2.96, 4.19) | | 2.45 (1.96, 3.09) | | 2.55 (1.97, 3.64) | | | 2.67 (2.00, 3.37) | | < 0.001 | 0.003 | | 0.774 |
| CRI-I | 4.56 (3.96, 5.19) | | 3.45 (2.96, 4.09) | | 3.55 (2.97, 4.64) | | | 3.67 (3.00, 4.37) | | < 0.001 | 0.003 | | 0.774 |
| CRI-II | 3.11 (2.65, 3.61) | | 2.29 (1.82, 2.83) | | 2.28 (1.84, 3.33) | | | 2.42 (1.89, 3.02) | | < 0.001 | 0.007 | | 0.886 |
| **Diabetes related biomarkers** | | |  | |  | | |  | |  |  | |  |
| GLU (mmol/L) | 6.65 (5.35, 9.44) | | 5.34 (4.66, 6.64) | | 6.66 (5.09, 10.18) | | | 5.02 (4.58, 5.90) | | < 0.001 | < 0.001 | | < 0.001 |
| TyG | 9.32 (8.94, 9.75) | | 8.5 (8.21, 8.84) | | 8.7 (8.27, 9.22) | | | 8.61 (8.24, 8.97) | | < 0.001 | 0.006 | | 0.14 |
| **Renal function indicators** | | |  | |  | | |  | |  |  | |  |
| Urea (mmol/L) | 5.6 (4.6, 6.9) | | 5.7 (4.5, 6.72) | | 6.8 (4.7, 9) | | | 5.5 (4.6, 6.7) | | 0.168 | 0.809 | | 0.002 |
| CREA (μmol/L) | 65.2 (55.6, 76.2) | | 62.5 (51.8, 73.6) | | 66.1 (50.9, 82.7) | | | 61.1 (50.9, 71. 7) | | < 0.001 | 0.161 | | 0.226 |
| UCR | 0.08 (0.07, 0.11) | | 0.08 (0.07, 0.1) | | 0.09 (0.08, 0.12) | | | 0.09 (0.07, 0.11) | | 0.047 | 0.066 | | 0.146 |
| UA (μmol/L) | 328 (262, 406) | | 300 (246.5, 357.5) | | 303 (248.6, 393) | | | 299 (249, 355) | | < 0.001 | 0.994 | | 0.415 |
| **Ion** |  |  | |  | |  |  | |  | |  |  |  |
| K (mmol/L) | 3.79 (3.51, 4.03) | | 3.8 (3.56, 3.99) | | 3.72 (3.53, 4.01) | | | 3.87 (3.64, 4.09) | | < 0.001 | < 0.001 | | 0.049 |
| NA (mmol/L) | 139.7 (138.0, 141.2) | | 140.4 (138.3, 142) | | 138.0 (135.3, 140) | | | 141.0 (139.0, 142.2) | | < 0.001 | 0.005 | | < 0.001 |
| Cl (mmol/L) | 105.0 (102.0, 106.8) | | 106.0 (104.0, 108.0) | | 105.0 (102.0, 107.2) | | | 106.7 (105.0, 108.1) | | < 0.001 | 0.041 | | 0.005 |
| CO2 (mmol/L) | 24.3 (22.5, 26.1) | | 24.4 (22.8, 26.1) | | 22.3 (19.5, 24.3) | | | 25 (23.4, 26.5) | | 0.004 | 0.002 | | < 0.001 |
| Ca (mmol/L) | 2.29 (2.23, 2.37) | | 2.2 (2.14, 2.28) | | 2.23 (2.13, 2.29) | | | 2.25 (2.18, 2.32) | | < 0.001 | < 0.001 | | 0.047 |
| P (mmol/L) | 1.03 (0.92, 1.17) | | 1.05 (0.93, 1.18) | | 1.03 (0.85, 1.15) | | | 1.11 (0.98, 1.23) | | < 0.001 | < 0.001 | | 0.15 |
| Mg (mmol/L) | 0.86 (0.81, 0.91) | | 0.84 (0.8, 0.89) | | 0.83 (0.76, 0.88) | | | 0.87 (0.83, 0.91) | | 0.04 | < 0.001 | | < 0.001 |
| **Liver function related indicators** | | |  | |  | | |  | |  |  | |  |
| TBIL (μmol/L) | 14.5 (10.9, 18.3) | | 14.9 (11.0, 20.1) | | 17.4 (13.4, 23.4) | | | 14.0 (11.1, 17.9) | | 0.774 | 0.08 | | 0.006 |
| DBIL (μmol/L) | 2.5 (1.8, 3.4) | | 3 (2.2, 4.3) | | 3.5 (2.1, 6.3) | | | 2.7 (2.0, 3.6) | | 0.103 | < 0.001 | | 0.001 |
| IBIL (μmol/L) | 11.6 (9.0, 15.2) | | 11.6 (8.5, 15.9) | | 13 (9.6, 18.2) | | | 11.1 (8.7, 14.7) | | 0.417 | 0.481 | | 0.031 |
| ALT (U/L) | 19 (13, 27) | | 17 (11, 25) | | 17 (13, 26) | | | 19 (13, 28) | | 0.899 | 0.001 | | 0.682 |
| AST (U/L) | 21 (19, 27) | | 21 (17, 25) | | 26 (22, 40) | | | 22 (18, 27) | | 0.812 | 0.142 | | < 0.001 |
| AAR | 1.11 (0.88, 1.54) | | 1.22 (0.96, 1.58) | | 1.57 (1.24, 2.17) | | | 1.14 (0.86, 1.47) | | 0.733 | < 0.001 | | < 0.001 |
| GGT (U/L) | 28 (20, 43) | | 21 (15, 32) | | 33 (19, 86) | | | 22 (16, 34) | | < 0.001 | 0.193 | | 0.003 |
| ALP (U/L) | 92 (76, 109) | | 83 (69, 98) | | 97 (67, 120) | | | 80 (67, 94) | | < 0.001 | 0.025 | | 0.014 |
| CHE (U/mL) | 8.81 ± 1.36 | | 7.18 ± 1.31 | | 6.6 ± 1.98 | | | 7.68 ± 1.43 | | < 0.001 | < 0.001 | | < 0.001 |
| TP (g/L) | 70.2 (66.8, 74.3) | | 64.5 (61.1, 68.6) | | 69.1 (65.1, 71.6) | | | 66.1 (62.1, 70.9) | | < 0.001 | < 0.001 | | 0.051 |
| ALB (g/L) | 41.5 (39.4, 43.8) | | 38.8 (36.5, 40.9) | | 37.7 (34.1, 39.9) | | | 39.9 (37.5, 42.8) | | < 0.001 | < 0.001 | | < 0.001 |
| GLB (g/L) | 28.5 (25.9, 32.1) | | 25.7 (23, 28.8) | | 30.2 (27.6, 34.4) | | | 26.0 (23.5, 29.1) | | < 0.001 | 0.311 | | < 0.001 |
| AGR | 1.44 (1.28, 1.63) | | 1.49 (1.32, 1.71) | | 1.29 (1.03, 1.39) | | | 1.54 (1.40, 1.71) | | < 0.001 | 0.018 | | < 0.001 |
| **Myocardial injury marker** | | |  | |  | | |  | |  |  | |  |
| CK (U/L) | 74 (55, 107) | | 70 (49, 96) | | 92 (60, 159) | | | 72 (53, 100) | | 0.16 | 0.282 | | 0.01 |
| CK-MB (U/L) | 13 (10, 15) | | 12 (10, 15) | | 13 (10, 16) | | | 12 (10, 14) | | 0.003 | 0.332 | | 0.04 |
| LDH (U/L) | 196 (171, 223) | | 187 (164, 220) | | 229 (189, 285) | | | 184 (161, 211) | | < 0.001 | 0.059 | | < 0.001 |
| **Coagulative markers** | | |  | |  | | |  | |  |  | |  |
| PT (s) | 10.9 (10.5, 11.5) | | 11.3 (10.9, 11.9) | | 12.7 (11.7, 13.6) | | | 11.0 (10.5, 11.4) | | 0.327 | < 0.001 | | < 0.001 |
| PTA (%) | 101 (93, 108) | | 95 (88, 101) | | 80 (72, 92) | | | 100 (93, 107) | | 0.497 | < 0.001 | | < 0.001 |
| INR | 0.99 (0.93, 1.02) | | 1.00 (0.99, 1.07) | | 1.10 (1.01, 1.2) | | | 1.00 (0.96, 1.04) | | < 0.001 | 0.002 | | < 0.001 |
| APTT (s) | 30.5 (28.7, 33.1) | | 30.7 (28.5, 33.0) | | 29.8 (27.4, 32.3) | | | 31.2 (29.0, 33.2) | | 0.152 | 0.087 | | 0.03 |
| FIB (g/L) | 3.08 (2.71, 3.51) | | 2.96 (2.61, 3.37) | | 3.72 (3.12, 4.95) | | | 2.89 (2.51, 3.24) | | < 0.001 | 0.011 | | < 0.001 |
| TT (s) | 13.7 (13.2, 14.6) | | 14.2 (13.5, 15.1) | | 14.1 (13, 15.2) | | | 14.3 (13.5, 15.0) | | < 0.001 | 0.266 | | 0.746 |
| DD (μg/mL) | 0.34 (0.20, 0.60) | | 0.44 (0.25, 0.84) | | 1.44 (0.82, 3.72) | | | 0.37 (0.22, 0.63) | | 0.143 | 0.002 | | < 0.001 |
| FDP (μg/mL) | 0.90 (0.59, 1.44) | | 1.12 (0.66, 2.16) | | 2.93 (1.81, 7.65) | | | 1.01 (0.60, 1.72) | | 0.024 | 0.144 | | < 0.001 |

AIS, acute ischemic stroke; HTN, hypertension; AF, atrial fibrillation; CHD, coronary heart disease; DM, diabetes mellitus; HR, heart rate; SaO2, oxygen saturation in arterial blood; SBP, systolic blood pressure; DBP, diastolic blood pressures; BMI, body mass index; WBC, white blood cell; NEU, neutrophil; LYM, lymphocyte; MON, monocyte, NLR, neutrophil to lymphocyte ratio; LMR, lymphocyte to monocyte ratio; MHR, monocyte to high-density lipoprotein-cholesterol ratio; NHR, neutrophil to high-density lipoprotein-cholesterol ratio; SII, systemic immune-inflammation index, SIRI, system inflammation response index; MII-1,multi-inflammatory index-1; MII-2, multi-inflammatory index-2; MII-3, multi-inflammatory index-3; RPR, red blood cell distribution width to platelet ratio; CRP, C-reaction protein. RBC, red blood cell; HGB, hemoglobin; HCT, hematocrit; MCV, mean corpuscular volume; MCH, Mean corpuscular hemoglobin; MCHC, mean corpuscular hemoglobin concentration; RDWSD, red blood cell distribution width standard deviation; RDWCV, red blood cell distribution width coefficient of variation; TC, total cholesterol; TG, total triglyceride; HDL-C, high-density lipoprotein-cholesterol; LDL-C, low-density lipoprotein cholesterol; AIP, atherogenic index of plasma; LCI, lipoprotein combine index; AC, atherogenic coefficient; CRI-I, Castelli’s index-I; CRI-II, Castelli’s index-II; non-HDL, non-high density lipoprotein-cholesterol; GLU, glucose; TyG, triglyceride-glucose; CREA, creatinine; UCR, urea to creatinine ratio; UA, uric acid; K, potassium; Na, sodium; Cl, chlorine; CO2, carbon dioxide; Ca, calcium; P, phosphorus; Mg, magnesium; TBIL, total bilirubin; DBIL, direct bilirubin; IBIL, indirect bilirubin; ALT, alanine transaminase; AST, aspartate aminotransferase; AAR, aspartate aminotransferase to alanine transaminase ratio; GGT, γ glutamyl transpeptadase; ALP, alkaline phosphatase; CHE, cholinesterase; TP, total protein; ALB, albumin; G, globulin; AGR, albumin to globulin ratio; CK, creatine kinase; CK-MB, creatine kinase-MB; LDH, lactic dehydrogenase; PT, prothrombin time; PTA, prothrombin activity; INR, international normalized ratio; APTT, activated partial thromboplastin time; FIB, fibrinogen; TT, thrombin time; FDP, fibrin degradation products; DD, D-Dimer.
