# Supplementary material for: Using a k-means clustering to identify novel phenotypes of acute ischemic stroke and development of its Clinlabomics models
Source: Front Neurol. 2024 Mar 27;15:1366307. doi: 10.3389/fneur.2024.1366307 (PMC11004235; doi:10.3389/fneur.2024.1366307)
Supplement: Supplementary file 2 [file Table_2.docx]

Supplementary Table 2 Characteristics of three clusters in the validation dataset based on the k-means clustering analysis

| Variables | Total (n = 507) | | Clusters | | | *P* |
| --- | --- | --- | --- | --- | --- | --- |
|  |  |  | Cluster A (n = 251) | Cluster B (n = 213) | Cluster C (n = 43) |  |
| Demographic characteristics | | |  |  |  |  |
| Age | 60 (53, 71) | | 62 (55, 72) | 58 (52, 66) | 61 (52, 73) | < 0.001^a^, 0.424, 0.186 |
| Gender |  | |  |  |  |  |
| Female (%) | 152 (30) | | 90 (36) | 47 (22) | 15 (35) | 0.002 ^a^, 1, 0.111 |
| Male (%) | 355 (70) | | 161 (64) | 166 (78) | 28 (65) |  |
| Nationality |  | |  |  |  |  |
| Han (%) | 458 (90) | | 227 (90) | 197 (92) | 34 (79) | 0.537, 0.038^b^, 0.02^c^ |
| Minority (%) | 49 (10) | | 24 (10) | 16 (8) | 9 (21) |  |
| Marriage |  | |  |  |  |  |
| Married (%) | 491 (97) | | 241 (96) | 210 (99) | 40 (93) | 0.164, 0.414, 0.061 |
| Other status (%) | 16 (3) | | 10 (4) | 3 (1) | 3 (7) |  |
| Education |  | |  |  |  |  |
| High school diploma or higher (%) | 153 (30) | | 69 (27) | 76 (36) | 8 (19) | 0.072, 0.3, 0.046 ^c^ |
| Others (%) | 354 (70) | | 182 (73) | 137 (64) | 35 (81) |  |
| Clinical classification and scores | | |  |  |  |  |
| TOAST | |  |  |  |  |  |
| LAA (%) | 199 (39) | | 85 (34) | 93 (44) | 21 (49) | 0.093, 0.014 ^b^, 0.066 |
| SAO (%) | 82 (16) | | 48 (19) | 33 (15) | 1 (2) |  |
| Others (%) | 226 (45) | | 118 (47) | 87 (41) | 21 (49) |  |
| Scales |  | |  |  |  |  |
| NIHSS | 2 (0, 5) | | 2 (0, 4) | 2 (0, 5) | 11 (4, 17) | 0.814, < 0.001 ^b^, < 0.001 ^c^ |
| GCS | 15 (15, 15) | | 15 (15, 15) | 15 (15, 15) | 13 (7, 15) | 0.729, < 0.001 ^b^, < 0.001 ^c^ |
| mRS |  | |  |  |  |  |
| 0-2 (%) | 339 (67) | | 187 (75) | 140 (66) | 12 (28) | 0.05, < 0.001 ^b^, < 0.001 ^c^ |
| 3-6 (%) | 168 (33) | | 64 (25) | 73 (34) | 31 (72) |  |
| Previous history | | |  |  |  |  |
| HTN |  | |  |  |  |  |
| No (%) | 224 (44) | | 109 (43) | 89 (42) | 26 (60) | 0.793, 0.057, 0.038 ^c^ |
| Yes (%) | 283 (56) | | 142 (57) | 124 (58) | 17 (40) |  |
| AF |  | |  |  |  |  |
| No (%) | 498 (98) | | 247 (98) | 209 (98) | 42 (98) | 1, 0.549, 1 |
| Yes (%) | 9 (2) | | 4 (2) | 4 (2) | 1 (2) |  |
| CHD |  | |  |  |  |  |
| No (%) | 487 (96) | | 236 (94) | 211 (99) | 40 (93) | 0.009 ^a^, 0.734, 0.035 ^c^ |
| Yes (%) | 20 (4) | | 15 (6) | 2 (1) | 3 (7) |  |
| DM |  | |  |  |  |  |
| No (%) | 434 (86) | | 215 (86) | 180 (85) | 39 (91) | 0.829, 0.516, 0.415 |
| Yes (%) | 73 (14) | | 36 (14) | 33 (15) | 4 (9) |  |
| Unhealthy habits | | |  |  |  |  |
| Smoking |  | |  |  |  |  |
| No (%) | 397 (78) | | 204 (81) | 162 (76) | 31 (72) | 0.208, 0.237, 0.722 |
| Yes (%) | 110 (22) | | 47 (19) | 51 (24) | 12 (28) |  |
| Drinking |  | |  |  |  | 0.402, 0.7, 0.486 |
| No (%) | 479 (94) | | 239 (95) | 198 (93) | 42 (98) |  |
| Yes (%) | 28 (6) | | 12 (5) | 15 (7) | 1 (2) |  |
| MRI location | | |  |  |  |  |
| Lesions |  | |  |  |  |  |
| One site (%) | 213 (42) | | 119 (47) | 87 (41) | 7 (16) | 0.185, < 0.001 ^b^, 0.004 ^c^ |
| Multiple sites (%) | 294 (58) | | 132 (53) | 126 (59) | 36 (84) |  |
| Carotid artery ultrasound | | |  |  |  |  |
| IMT: Right |  | |  |  |  |  |
| ≤1.0 mm (%) | 391 (77) | | 190 (76) | 165 (77) | 36 (84) | 0.736, 0.338, 0.479 ^c^ |
| >1.0 mm (%) | 116 (23) | | 61 (24) | 48 (23) | 7 (16) |  |
| IMT: Left |  | |  |  |  |  |
| ≤1.0 mm (%) | 396 (78) | | 192 (76) | 166 (78) | 38 (88) | 0.797, 0.123, 0.179 |
| >1.0 mm (%) | 111 (22) | | 59 (24) | 47 (22) | 5 (12) |  |
| CP |  | |  |  |  |  |
| No (%) | 150 (30) | | 74 (29) | 68 (32) | 8 (19) | 0.64, 0.199, 0.119 |
| Yes (%) | 357 (70) | | 177 (71) | 145 (68) | 35 (81) |  |
| VP |  | |  |  |  |  |
| None (%) | 151 (30) | | 74 (29) | 68 (32) | 9 (21) | 0.762, 0.052, 0.022 ^c^ |
| SP (%) | 37 (7) | | 19 (8) | 18 (8) | 0 (0) |  |
| VP (%) | 319 (63) | | 158 (63) | 127 (60) | 34 (79) |  |
| CS |  | |  |  |  |  |
| No (%) | 433 (85) | | 219 (87) | 178 (84) | 36 (84) | 0.321, 0.699, 1 |
| Yes (%) | 74 (15) | | 32 (13) | 35 (16) | 7 (16) |  |
| Non-invasive physiological indices | | |  |  |  |  |
| HR (bpm) | 79 (70, 88) | | 78 (69, 83) | 80 (70, 91) | 81 (70, 93) | 0.003 ^a^, 0.088, 0.659 |
| SBP (mmHg) | 139 (125, 151) | | 136 (122, 150) | 141 (130, 157) | 130 (117, 149) | 0.002 ^a^, 0.403, 0.008 ^c^ |
| DBP (mmHg) | 83 ± 14 | | 79 ± 13 | 88 ± 13 | 80 ± 13 | < 0.001 ^a^, 0.761, < 0.001 ^c^ |
| SaO2 (%) | 95 (94, 96) | | 95 (94, 96) | 95 (95, 96) | 96 (95, 97) | 0.048 ^a^, 0.044 ^b^, 0.327 |
| Individual characteristics | | |  |  |  |  |
| Weight (Kg) | 68 (60, 75) | | 65 (58, 73) | 70 (65, 77) | 65 (55, 70) | < 0.001 ^a^, 0.186, < 0.001 ^c^ |
| Height (cm) | 168 (160, 173) | | 167 (160, 172) | 170 (164, 174) | 167 (160, 170) | 0.001 ^a^, 0.928, 0.049 ^c^ |
| BMI (Kg/m^2) | 24.16 (22.08, 26.04) | | 23.44 (21.43, 25.71) | 24.86 (23.05, 27.24) | 23.12 (20.2, 25.16) | < 0.001 ^a^, 0.066, < 0.001 ^c^ |
| Inflammatory biomarkers | | |  |  |  |  |
| WBC (10^9^/L) | 6.82 (5.50, 8.30) | | 6.08 (4.90, 7.40) | 7.42 (6.22, 8.80) | 11.00 (9.00, 13.40) | < 0.001 ^a^, < 0.001 ^b^, < 0.001 ^c^ |
| NEU (10^9^/L) | 4.44 (3.34, 5.90) | | 3.75 (2.99, 4.80) | 4.77 (3.96, 6.00) | 9.61 (7.36, 11.86) | < 0.001 ^a^, < 0.001 ^b^, < 0.001 ^c^ |
| LYM (10^9^/L) | 1.53 (1.21, 2.09) | | 1.48 (1.25, 2.03) | 1.77 (1.33, 2.26) | 1.01 (0.58, 1.23) | 0.001 ^a^, < 0.001 ^b^, < 0.001 ^c^ |
| MON (10^9^/L) | 0.43 (0.32, 0.54) | | 0.41 (0.31, 0.51) | 0.44 (0.34, 0.53) | 0.58 (0.39, 0.72) | 0.014 ^a^, < 0.001 ^b^, < 0.001 ^c^ |
| NLR | 2.64 (1.79, 4.37) | | 2.30 (1.71, 3.38) | 2.62 (1.79, 4.29) | 10.30 (8.14, 13.96) | 0.015 ^a^, < 0.001 ^b^, < 0.001 ^c^ |
| LMR | 3.79 (2.72, 5.12) | | 3.80 (2.92, 5.24) | 4.07 (3.19, 5.32) | 1.88 (1.20, 2.13) | 0.252, < 0.001 ^b^, < 0.001 ^c^ |
| MHR | 0.45 (0.31, 0.58) | | 0.42 (0.30, 0.54) | 0.45 (0.33, 0.58) | 0.57 (0.44, 0.76) | 0.021 ^a^, < 0.001 ^b^, 0.002 ^c^ |
| NHR | 4.63 (3.29, 6.44) | | 3.75 (2.79, 5.52) | 4.99 (3.70, 6.57) | 10.2 (7.26, 13.95) | 0.001 ^a^, < 0.001 ^b^, < 0.001 ^c^ |
| SII (10^9^/L) | 535 (339, 883) | | 445 (286, 677) | 551 (380, 875) | 2167 (1533, 3051) | 0.001 ^a^, < 0.001 ^b^, < 0.001 ^c^ |
| SIRI (10^9^/L) | 1.15 (0.70, 1.90) | | 0.97 (0.61, 1.50) | 1.17 (0.76, 1.80) | 5.78 (3.77, 7.76) | 0.001 ^a^, < 0.001 ^b^, < 0.001 ^c^ |
| MII-1 | 12.08 (4.65, 22.41) | | 11.02 (2.90, 18.98) | 10.95 (5.32, 19.34) | 66.24 (39.58, 217.37) | 0.152, < 0.001 ^b^, < 0.001 ^c^ |
| MII-2 | 554 (195, 1011) | | 566 (142, 934) | 475 (215, 866) | 1829 (567, 5548) | 0.573, < 0.001 ^b^, < 0.001 ^c^ |
| MII-3 | 2272 (904, 4725) | | 1869 (533, 3584) | 2268 (1085, 4114) | 15782 (6608, 47683) | 0.008 ^a^, < 0.001 ^b^, < 0.001 ^c^ |
| RPR | 0.07 (0.06, 0.08) | | 0.07 (0.06, 0.09) | 0.06 (0.05, 0.07) | 0.06 (0.05, 0.10) | < 0.001 ^a^, 0.354, 0.148 |
| CRP (mg/L) | 6.37 (1.48, 6.94) | | 6.94 (1.06, 6.94) | 4.89 (1.66, 6.94) | 6.94 (3.76, 26.16) | 0.748, < 0.001 ^b^, < 0.001 ^c^ |
| Red blood cell related parameters | | |  |  |  |  |
| RBC (10^12^/L) | 4.82 (4.44, 5.19) | | 4.68 (4.28, 5.02) | 5.05 (4.74, 5.36) | 4.56 (4.11, 4.86) | < 0.001 ^a^, 0.057, < 0.001 ^c^ |
| HGB (g/L) | 150 (138, 162) | | 145 (133, 155) | 158 (149, 168) | 135 (124, 151) | < 0.001 ^a^, 0.04 ^b^, < 0.001 ^c^ |
| HCT | 0.45 (0.41, 0.48) | | 0.44 (0.40, 0.47) | 0.47 (0.44, 0.50) | 0.41 (0.38, 0.45) | < 0.001 ^a^, 0.009 ^b^, < 0.001 ^c^ |
| MCV (fL) | 92.7 (89.8, 95.8) | | 93 (90.1, 96.0) | 92.5 (89.8, 95.7) | 91.6 (88.6, 94.2) | 0.597, 0.076, 0.152 |
| MCH (pg) | 31.1 (29.9, 32.1) | | 31.0 (29.6, 32.0) | 31.2 (30.3, 32.4) | 30.7 (29.0, 31.9) | 0.006 ^a^, 0.953, 0.131 |
| MCHC (g/L) | 334 (326, 343) | | 330 (324, 339) | 338 (330, 345) | 338 (327, 343) | < 0.001 ^a^, 0.025 ^b^, 0.353 |
| RDWCV (%) | 13.1 (12.6, 13.7) | | 13.1 (12.6, 13.8) | 13.0 (12.6, 13.5) | 13.4 (12.7, 14.3) | < 0.001 ^a^, 0.025 ^b^, 0.353 |
| Lipid parameters | | |  |  |  |  |
| TC (mmol/L) | 4.17 (3.44, 4.88) | | 3.61 (3.09, 4.26) | 4.82 (4.23, 5.58) | 3.65 (3.00, 4.23) | < 0.001 ^a^, 0.769, < 0.001 ^c^ |
| TG (mmol/L) | 1.46 (0.99, 1.98) | | 1.14 (0.90, 1.48) | 2.08 (1.58, 2.82) | 0.92 (0.76, 1.30) | < 0.001 ^a^, 0.014 ^b^, < 0.001 ^c^ |
| HDL-C (mmol/L) | 0.98 (0.85, 1.15) | | 0.99 (0.84, 1.16) | 0.96 (0.86, 1.12) | 0.99 (0.78, 1.10) | 0.396, 0.434, 0.762 |
| LDL-C (mmol/L) | 2.83 (2.17, 3.38) | | 2.35 (1.94, 2.84) | 3.34 (2.90, 3.82) | 2.45 (1.98, 2.93) | < 0.001 ^a^, 0.54, < 0.001 ^c^ |
| AIP | 0.18 ± 0.26 | | 0.06 ± 0.21 | 0.34 ± 0.23 | 0.04 ± 0.23 | < 0.001 ^a^, 0.462, < 0.001 ^c^ |
| LCI | 16.67 (8.54, 30.14) | | 10.14 (6.74, 15.68) | 33.18 (23.61, 50.16) | 9.63 (4.95, 15.65) | < 0.001 ^a^, 0.476, < 0.001 ^c^ |
| non-HDL-C (mmol/L) | 3.20 (2.47, 3.84) | | 2.65 (2.13, 3.20) | 3.87 (3.37, 4.41) | 2.59 (2.13, 3.22) | < 0.001 ^a^, 0.789, < 0.001 ^c^ |
| AC | 3.23 (2.52, 3.97) | | 2.67 (2.18, 3.20) | 3.99 (3.52, 4.52) | 2.80 (2.17, 3.48) | < 0.001 ^a^, 0.347, < 0.001 ^c^ |
| CRI-I | 4.23 (3.52, 4.97) | | 3.67 (3.18, 4.20) | 4.99 (4.52, 5.52) | 3.80 (3.17, 4.48) | < 0.001 ^a^, 0.347, < 0.001 ^c^ |
| CRI-II | 2.83 (2.24, 3.46) | | 2.43 (1.93, 2.83) | 3.43 (2.96, 3.86) | 2.62 (2.01, 3.29) | < 0.001 ^a^, 0.082, < 0.001 ^c^ |
| Diabetes related biomarkers | | |  |  |  |  |
| GLU (mmol/L) | 6.20 (5.19, 7.81) | | 5.67 (4.94, 7.03) | 6.64 (5.52, 9.37) | 7.39 (6.46, 8.43) | < 0.001 ^a^, < 0.001 ^b^, 0.271 |
| TyG | 9.62 (9.14, 10.05) | | 9.27 (8.95, 9.66) | 10.03 (9.69, 10.57) | 9.25 (9.08, 9.79) | < 0.001 ^a^, 0.481, < 0.001 ^c^ |
| Renal function indicators | | |  |  |  |  |
| Urea (mmol/L) | 5.4 (4.4, 6.7) | | 5.3 (4.3, 6.6) | 5.6 (4.5, 6.7) | 5.6 (4.5, 7.5) | 0.19, 0.224, 0.568 |
| CREA (μmol/L) | 65.6 (56.1, 75.3) | | 63.3 (55.4, 72.4) | 69.3 (60.2, 81.2) | 57.7 (51.1, 69.1) | < 0.001 ^a^, 0.046 ^b^, < 0.001 ^c^ |
| UCR | 0.08 (0.07, 0.10) | | 0.09 (0.07, 0.10) | 0.08 (0.06, 0.09) | 0.10 (0.07, 0.12) | 0.011 ^a^, 0.026 ^b^, < 0.001 ^c^ |
| UA (μmol/L) | 304.0 (253.4, 369.4) | | 286.0 (245.5, 333.5) | 348.0 (288.9, 415.0) | 263.4 (196.1, 355.7) | < 0.001 ^a^, 0.522, < 0.001 ^c^ |
| Ion | | |  |  |  |  |
| K (mmol/L) | 3.70 (3.48, 3.94) | | 3.70 (3.48, 3.92) | 3.74 (3.52, 4.02) | 3.57 (3.45, 3.77) | 0.107, 0.094, 0.013 ^c^ |
| NA (mmol/L) | 137.4 (135.5, 138.9) | | 138.1 (136.8, 139.5) | 136.7 (134.9, 138.5) | 135.9 (132.1, 136.8) | < 0.001 ^a^, < 0.001 ^b^, 0.002 ^c^ |
| Cl (mmol/L) | 104.0 (101.7, 106.0) | | 104.9 (103.1, 106.6) | 103.1 (100.5, 104.9) | 102.0 (98.9, 104.4) | < 0.001 ^a^, < 0.001 ^b^, 0.09 |
| CO2 (mmol/L) | 24. 6 ± 3.0 | | 25.1 ± 2.7 | 24.1 ± 3.1 | 23.5 ± 3.8 | < 0.001 ^a^, 0.002 ^b^, 0.349 |
| Ca (mmol/L) | 2.27 (2.20, 2.33) | | 2.24 (2.18, 2.30) | 2.30 (2.25, 2.37) | 2.23 (2.14, 2.30) | < 0.001 ^a^, 0.206, < 0.001 ^c^ |
| P (mmol/L) | 1.05 ± 0.20 | | 1.06 ± 0.19 | 1.06 ± 0.21 | 0.96 ± 0.22 | 0.657, 0.007 ^b^, 0.014 ^c^ |
| Mg (mmol/L) | 0.86 (0.81, 0.91) | | 0.85 (0.81, 0.90) | 0.87 (0.82, 0.92) | 0.80 (0.76, 0.86) | 0.028 ^a^, < 0.001 ^b^, < 0.001 ^c^ |
| Liver function related indicators | | |  |  |  |  |
| TBIL (μmol/L) | 14.2 (10.4, 18.4) | | 14.8 (10.5, 18.8) | 13.1 (10.0, 16.8) | 15.8 (13.8, 20.5) | 0.037 ^a^, 0.023 ^b^, < 0.001 ^c^ |
| DBIL (μmol/L) | 2.9 (2.0, 3.9) | | 3.0 (2.2, 3.9) | 2.6 (1.8, 3.6) | 3.7 (3.0, 4.7) | 0.011 ^a^, 0.002 ^b^, < 0.001 ^c^ |
| IBIL (μmol/L) | 11.2 (8.0, 15.0) | | 11.5 (8.4, 15.2) | 10.5 (7.6, 14.1) | 12.5 (9.6, 16.5) | 0.075, 0.086, 0.007 ^c^ |
| ALT (U/L) | 19 (15, 27) | | 17 (13, 23) | 22 (16, 33) | 23 (18, 32) | < 0.001 ^a^, 0.004 ^b^, 0.891 |
| AST (U/L) | 23 (18, 27) | | 21 (18, 26) | 23 (19, 28) | 29 (23, 43) | 0.008 ^a^, < 0.001 ^b^, < 0.001 ^c^ |
| AAR | 1.14 (0.92, 1.50) | | 1.23 (1.00, 1.56) | 1.02 (0.82, 1.29) | 1.35 (1.06, 1.85) | < 0.001 ^a^, 0.079, < 0.001 ^c^ |
| GGT (U/L) | 27 (18, 42) | | 21 (15, 32) | 35 (22, 54) | 28 (20, 42) | < 0.001 ^a^, < 0.001 ^b^, 0.057 |
| ALP (U/L) | 88 (71, 108) | | 83 (69, 104) | 92 (75, 113) | 92 (80, 115) | 0.008 ^a^, 0.031 ^b^, 0.535 |
| CHE (U/mL) | 7.8 (6.94, 8.84) | | 7.59 (6.74, 8.59) | 8.24 (7.31, 9.18) | 7.69 (6.56, 9.00) | < 0.001 ^a^, 0.609, 0.074 |
| TP (g/L) | 68.6 ± 7.1 | | 66.1 ± 6.6 | 72.0 ± 6.4 | 66.7 ± 6.6 | < 0.001 ^a^, 0.593, < 0.001 ^c^ |
| ALB (g/L) | 39.5 (37.3, 42.2) | | 38.8 (36.7, 40.9) | 41.5 (39.3, 43.7) | 36.6 (34.1, 39.2) | < 0.001 ^a^, 0.016 ^b^, < 0.001 ^c^ |
| GLB (g/L) | 29.0 ± 4.9 | | 27.4 ± 4.4 | 30.7 ± 4.7 | 29.2 ± 5.4 | < 0.001 ^a^, 0.051, 0.047 ^c^ |
| AGR | 1.38 (1.25, 1.52) | | 1.40 (1.29, 1.54) | 1.35 (1.24, 1.48) | 1.27 (1.10, 1.40) | < 0.001 ^a^, < 0.001 ^b^, 0.013 ^c^ |
| Myocardial injury markers | | |  |  |  |  |
| CK (U/L) | 76 (53, 103) | | 70 (50, 95) | 83 (54, 103) | 95 (60, 149) | 0.008 ^a^, 0.003 ^b^, 0.044 ^c^ |
| CK-MB (U/L) | 11 (10, 13) | | 11 (10, 13) | 11 (10, 13) | 12 (10, 16) | 0.23, 0.046 ^b^, 0.158 |
| LDH (U/L) | 185 (157, 208) | | 180 (154, 202) | 182 (156, 205) | 221 (190, 266) | 0.529, < 0.001 ^b^, < 0.001 ^c^ |
| Coagulative markers | | |  |  |  |  |
| PT (s) | 11.4 (10.9, 11.7) | | 11.5 (11.0, 11.8) | 11.2 (10.7, 11.5) | 12.4 (11.8, 13.0) | < 0.001 ^a^, < 0.001 ^b^, < 0.001 ^c^ |
| PTA (%) | 95 (89, 100) | | 95 (88, 99) | 97 (93, 104) | 83 (77, 90) | < 0.001 ^a^, < 0.001 ^b^, < 0.001 ^c^ |
| INR | 1.05 (1.00, 1.07) | | 1.05 (1.01, 1.08) | 1.02 (0.97, 1.05) | 1.13 (1.07, 1.19) | < 0.001 ^a^, < 0.001 ^b^, < 0.001 ^c^ |
| APTT (s) | 30.8 (28.6, 32.4) | | 30.8 (29.0, 32.5) | 30.8 (28.8, 32.4) | 28.5 (26.2, 30.5) | 0.542, < 0.001 ^b^, < 0.001 ^c^ |
| FIB (g/L) | 3.01 (2.54, 3.30) | | 2.88 (2.43, 3.09) | 3.01 (2.69, 3.49) | 3.16 (2.46, 4.25) | < 0.001 ^a^, 0.007 ^b^, 0.218 |
| TT (s) | 14.6 (13.8, 15.3) | | 14.7 (14.3, 15.6) | 14.3 (13.4, 14.8) | 14.6 (13.8, 15.3) | < 0.001 ^a^, 0.078, 0.106 |
| DD (μg/mL) | 0.38 (0.23, 0.79) | | 0.38 (0.23, 0.76) | 0.31 (0.21, 0.59) | 1.42 (0.88, 2.37) | 0.022 ^a^, < 0.001 ^b^, < 0.001 ^c^ |
| FDP (μg/mL) | 1.47 (0.87, 2.41) | | 1.41 (0.88, 2.41) | 1.23 (0.80, 1.71) | 3.77 (2.01, 5.84) | 0.033 ^a^, < 0.001 ^b^, < 0.001 ^c^ |

AIS, acute ischemic stroke; TOAST, Trial of Org 10172 in Acute Stroke Treatment; LAA, large-artery atherosclerosis; SAO, small-artery occlusion; NIHSS, the National Institutes of Health Stroke Scale; GCS, Glasgow coma scale; mRS, modified Rankin scale; HTN, hypertension; AF, atrial fibrillation; CHD, coronary heart disease; DM, diabetes mellitus; IMT: intima-media thickness; CP, carotid plaque; VP, vulnerable plaque; SP, stable plaque; CS, carotid stenosis; HR, heart rate; SaO2, oxygen saturation in arterial blood; SBP, systolic blood pressure; DBP, diastolic blood pressures; BMI, body mass index; WBC, white blood cell; NEU, neutrophil; LYM, lymphocyte; MON, monocyte, NLR, neutrophil to lymphocyte ratio; LMR, lymphocyte to monocyte ratio; MHR, monocyte to high-density lipoprotein-cholesterol ratio; NHR, neutrophil to high-density lipoprotein-cholesterol ratio; SII, systemic immune-inflammation index, SIRI, system inflammation response index; MII-1,multi-inflammatory index-1; MII-2, multi-inflammatory index-2; MII-3, multi-inflammatory index-3; RPR, red blood cell distribution width to platelet ratio; CRP, C-reaction protein. RBC, red blood cell; HGB, hemoglobin; HCT, hematocrit; MCV, mean corpuscular volume; MCH, Mean corpuscular hemoglobin; MCHC, mean corpuscular hemoglobin concentration; RDWSD, red blood cell distribution width standard deviation; RDWCV, red blood cell distribution width coefficient of variation; TC, total cholesterol; TG, total triglyceride; HDL-C, high-density lipoprotein-cholesterol; LDL-C, low-density lipoprotein cholesterol; AIP, atherogenic index of plasma; LCI, lipoprotein combine index; AC, atherogenic coefficient; CRI-I, Castelli’s index-I; CRI-II, Castelli’s index-II; non-HDL, non-high density lipoprotein-cholesterol; GLU, glucose; TyG, triglyceride-glucose; CREA, creatinine; UCR, urea to creatinine ratio; UA, uric acid; K, potassium; Na, sodium; Cl, chlorine; CO2, carbon dioxide; Ca, calcium; P, phosphorus; Mg, magnesium; TBIL, total bilirubin; DBIL, direct bilirubin; IBIL, indirect bilirubin; ALT, alanine transaminase; AST, aspartate aminotransferase; AAR, aspartate aminotransferase to alanine transaminase ratio; GGT, γ glutamyl transpeptadase; ALP, alkaline phosphatase; CHE, cholinesterase; TP, total protein; ALB, albumin; G, globulin; AGR, albumin to globulin ratio; CK, creatine kinase; CK-MB, creatine kinase-MB; LDH, lactic dehydrogenase; PT, prothrombin time; PTA, prothrombin activity; INR, international normalized ratio; APTT, activated partial thromboplastin time; FIB, fibrinogen; TT, thrombin time; FDP, fibrin degradation products; DD, D-Dimer; ^a^, comparison between cluster A and B with p < 0.05; ^b^, comparison between cluster A and C with p < 0.05; ^c^, comparison between cluster B and C with p < 0.05.
