# Supplementary figures and images for: Using a k-means clustering to identify novel phenotypes of acute ischemic stroke and development of its Clinlabomics models
Source: Front Neurol. 2024 Mar 27;15:1366307. doi: 10.3389/fneur.2024.1366307 (PMC11004235; doi:10.3389/fneur.2024.1366307)

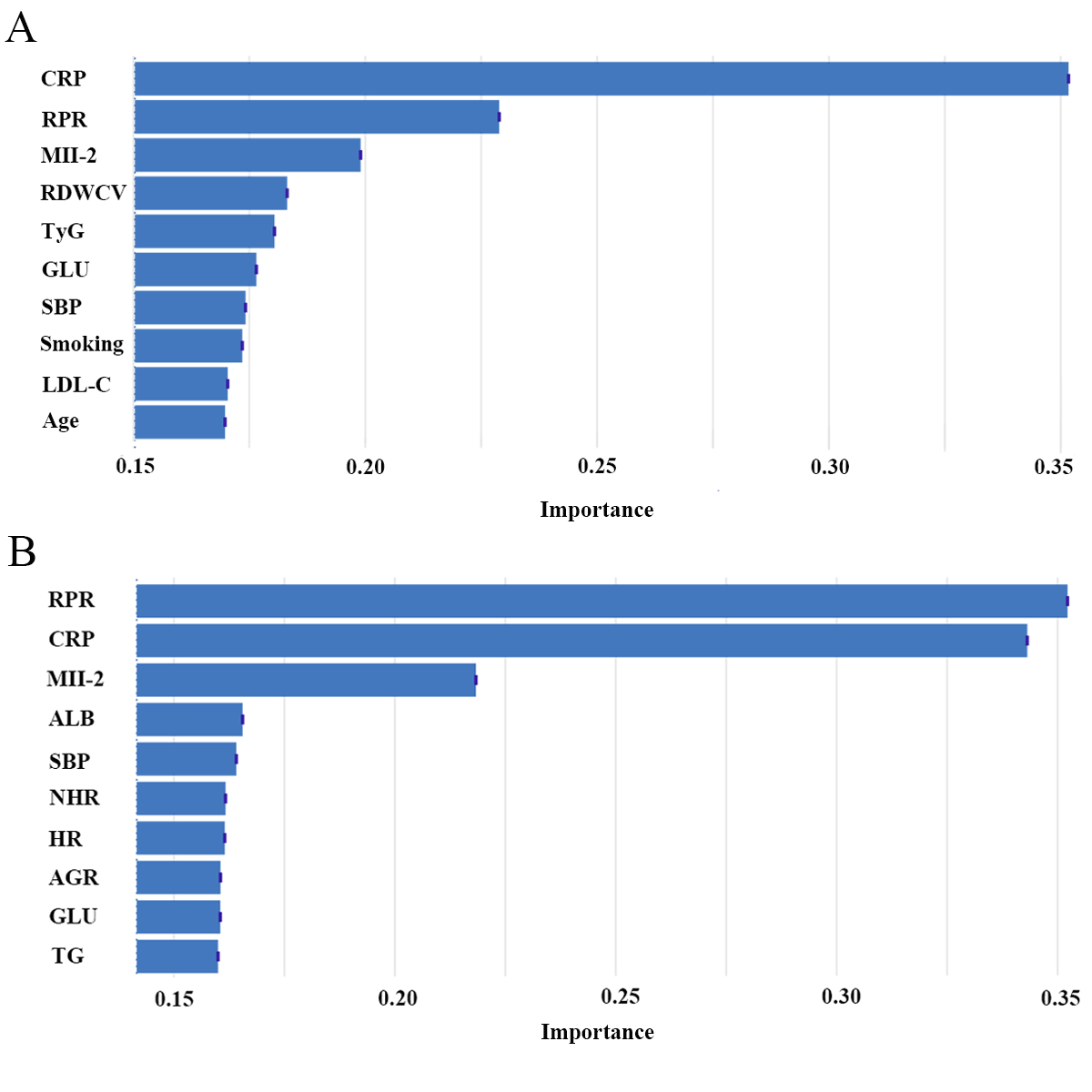

Supplement: Supplementary file 3 [file Image_1.TIF]
